# Inhibition of the transcriptional repressor complex Bcl-6/BCoR induces endothelial sprouting but does not promote tumor growth

**DOI:** 10.18632/oncotarget.13477

**Published:** 2016-11-21

**Authors:** Elisabeth Buchberger, Dietmar Payrhuber, Miriam El Harchi, Branislav Zagrapan, Katharina Scheuba, Anna Zommer, Edina Bugyik, Balazs Dome, Julia Barbara Kral, Waltraud Cornelia Schrottmaier, Gernot Schabbauer, Peter Petzelbauer, Marion Gröger, Martin Bilban, Christine Brostjan

**Affiliations:** ^1^ Department of Surgery, Medical University of Vienna, General Hospital, Vienna, Austria; ^2^ Comprehensive Cancer Center, Medical University of Vienna, Vienna, Austria; ^3^ First Department of Pathology and Experimental Cancer Research, Semmelweis University, Budapest, Hungary; ^4^ Department of Thoracic Surgery, Semmelweis University - National Institute of Oncology, Budapest, Hungary; ^5^ Hungarian Academy of Sciences Postdoctoral Research Programme, Budapest, Hungary; ^6^ Department of Thoracic Surgery, Medical University of Vienna, Vienna, Austria; ^7^ Department of Biomedical Imaging and Image-guided Therapy, Medical University of Vienna, Vienna, Austria; ^8^ National Koranyi Institute of Pulmonology, Budapest, Hungary; ^9^ Institute of Physiology, Center of Physiology and Pharmacology, Medical University of Vienna, Vienna, Austria; ^10^ Department of Dermatology, Medical University of Vienna, General Hospital, Vienna, Austria; ^11^ Core Facility Imaging, Medical University of Vienna, Vienna, Austria; ^12^ Department of Laboratory Medicine, Medical University of Vienna, General Hospital, Vienna, Austria; ^13^ Core Facility Genomics, Medical University of Vienna, Vienna, Austria

**Keywords:** 79-6, Bcl-6, BCoR, colorectal carcinoma, vascular sprouting

## Abstract

The oncogenic potential of the transcriptional repressor Bcl-6 (B-cell lymphoma 6) was originally discovered in non-Hodgkin patients and the soluble Bcl-6 inhibitor 79-6 was developed to treat diffuse large B-cell lymphomas with aberrant Bcl-6 expression. Since we found Bcl-6 and its co-repressor BCoR (Bcl-6 interacting co-repressor) to be regulated in human microvascular endothelium by colorectal cancer cells, we investigated their function in sprouting angiogenesis which is central to tumor growth. Based on Bcl-6/BCoR gene silencing we found that the transcriptional repressor complex in fact constitutes an endogenous inhibitor of vascular sprouting by supporting the stalk cell phenotype: control of Notch target genes (HES1, HEY1, DLL4) and cell cycle regulators (cyclin A and B1). Thus, when endothelial cells were transiently transfected with Bcl-6 and/or BCoR siRNA, vascular sprouting was prominently induced. Comparably, when the soluble Bcl-6 inhibitor 79-6 was applied in the mouse retina model of physiological angiogenesis, endothelial sprouting and branching were significantly enhanced. To address the question whether clinical treatment with 79-6 might therefore have detrimental therapeutic effects by promoting tumor angiogenesis, mouse xenograft models of colorectal cancer and diffuse large B-cell lymphoma were tested. Despite a tendency to increased tumor vessel density, 79-6 therapy did not enhance tumor expansion. In contrast, growth of colorectal carcinomas was significantly reduced which is likely due to a combined 79-6 effect on cancer cells and tumor stroma. These findings may provide valuable information regarding the future clinical development of Bcl-6 inhibitors.

## INTRODUCTION

Bcl-6 (B-cell lymphoma 6) is a transcriptional repressor which belongs to the class of zinc finger proteins and functions as a dimer [1–3]. A number of Bcl-6 interaction partners has been identified, among those the co-repressors SMRT, NCoR, BCoR and histone deacetylases (HDACs) [4, 5]. Bcl-6 is subject to regulation at the transcriptional and post-transcriptional level. Two mRNA variants were initially described (V1 and V2) which differ in their promoter start sites but encode identical Bcl-6 proteins [6, 7]. Of note, V2 transcript was found to be restricted to the nucleus and hence not translated.

Distinct from SMRT and NCoR, the Bcl-6 interacting co-repressor (BCoR) selectively recognizes Bcl-6 but does not interact with other Bcl-6 family members like BAZF [8]. Furthermore, BCoR competes with NCoR and SMRT for Bcl-6 binding. It recruits class I as well as class II HDACs thereby enhancing repression of Bcl-6 target genes. Four major isoforms of BCoR have been identified based on alternative splicing of exons 5 and 8. These isoforms are equally potent in promoting Bcl-6 activity but differ in their interaction with other transcription factors such as AF9 [9]. The BCoR variant lacking exon 8A but including exon 5 was most prominently expressed in human leukocytes. Of note, a substantially shorter isoform (BCoR-S) was reported which results from aberrant intron 4 processing and a premature stop signal and seems to lack transcriptional co-repressor activity [8]. The fact that BCoR can bind to transcription factors other than Bcl-6 may explain why aberrant BCoR expression [10] leads to pathologies distinct from Bcl-6 deficiency and points to a particular role of BCoR in embryonal development [11–13].

Of note, Bcl-6 was originally discovered in non-Hodgkin lymphomas where chromosomal translocations led to aberrant Bcl-6 expression [14, 15]. Functions of Bcl-6 have primarily been characterized in leukocytes, as Bcl-6 knock-out mice showed severe defects in germinal center formation and Th2 type hyperimmune responses [16, 17]. Bcl-6 was identified to control affinity maturation and proliferation of B-cells, cytokine and chemokine expression of T helper cells and macrophages [18]. Bcl-6 is known to positively or negatively affect cell proliferation depending on the cell type [19–21]. Cell cycle regulators controlled by Bcl-6 in a direct or indirect manner include p21 [22], p27, cyclin D1 and D2 [23, 24] and cyclin A2 [25].

BAZF (Bcl-6 associated zinc finger protein) is a close homolog of Bcl-6 and reportedly recognizes identical DNA elements [26, 27]. It can form heterodimers with Bcl-6 and the transcriptional repressor activity of BAZF was proposed to require Bcl-6 interaction for recruitment of histone deactylases and mSin3A [28]. With respect to endothelial cells, BAZF was recently discovered to play a central role in blood vessel formation by regulating the cross-talk between VEGF and Notch signaling [29]. Endothelial sprouting involves a migrating tip cell which is characterized by high VEGF and low Notch signaling [30]. In contrast, the adjacent stalk cells are defined by high Notch and low VEGF signaling, and proliferate to support sprout elongation. VEGF was found to induce BAZF expression via mRNA stabilization [31]. The binding of BAZF to the Notch signaling factor CBF-1 (C-promoter binding factor 1) resulted in CBF-1 degradation and the down-regulation of Notch signaling, thereby promoting VEGF-induced tip cell formation and angiogenic sprouting [29]. Since Ohnuki *et al.* did not detect Bcl-6 mRNA expression in this setting, the authors proposed the pro-angiogenic BAZF function to be independent of Bcl-6. Of note, Bcl-6 expression in endothelial cells has previously been reported to contribute to the anti-inflammatory effects of peroxisome proliferator-activated receptor-delta activation [32]. The association of Bcl-6 with co-repressors such as BCoR has not been investigated in endothelial cells to date.

The current study was initiated when analyzing the impact of colorectal cancer derived stimuli on the gene expression profile of human microvessel endothelial cells (ECs). We found BCoR mRNA to be prominently induced in endothelial cells in response to tumor signals. Based on this observation we investigated in detail the presence of BCoR and Bcl-6 transcript variants in ECs, the regulation of their gene expression at the mRNA and protein level, and their function in sprouting angiogenesis. Furthermore, the impact of Bcl-6 inhibition on microvessel density and tumor growth was addressed, since soluble Bcl-6 inhibitors have been developed for clinical cancer therapy [33].

## RESULTS

### Bcl-6 and BCoR mRNA is expressed in endothelial cells and further induced by tumor-derived stimuli

**Figure 1:**
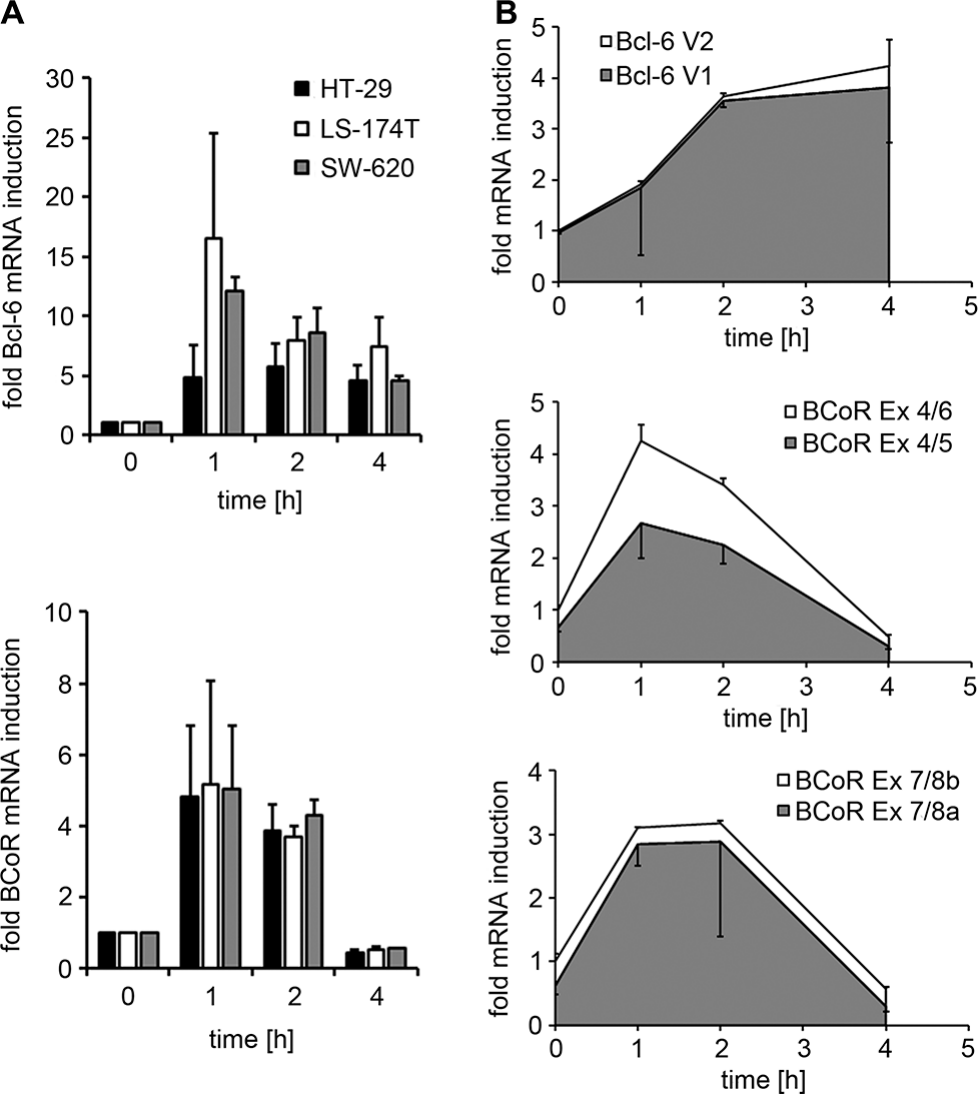
Endothelial Bcl-6/BCoR mRNA expression in response to tumor-derived stimuli (**A**) Bcl-6/BCoR mRNA levels were determined in ECs exposed to conditioned medium from three different colorectal cancer cell lines by real-time PCR using pan-reactive primer sets and are given in relation to untreated control. (**B**) The ratio of Bcl-6 V1/V2 splice variants and of BCoR transcripts with/without exon 5 or exon 8a was investigated in ECs treated with HT-29 supernatant for 1–4 hours. Specific primer sets were used to distinguish splice variants and the increase in mRNA levels was determined in relation to untreated control. The relative proportion of splice variants within the total amount of transcripts is illustrated by the colors grey and white. The results represent mean values and standard deviations of 2–3 independent experiments.

In a microarray screen conducted to identify endothelial genes regulated in response to tumor signals, BCoR transcripts were found to be 3.5-fold induced in ECs stimulated with conditioned medium from HT-29 colon carcinoma cells (Supplementary Table S1). In contrast, the microarray analysis did not detect mRNA changes for Bcl-6 or competing Bcl-6 co-repressors NCoR and SMRT. However, subsequent transcript analysis by quantitative real-time PCR demonstrated a rapid, 5- to 17-fold induction of both Bcl-6 and BCoR mRNA in ECs within 1 h of stimulation with tumor-derived signals (Figure 1A). The effect was observed using three different colon carcinoma cell lines (HT-29, LS174T and SW620) and various breast cancer cell lines (data not shown). Endothelial activation by tumor cell supernatant resulted in peak levels of Bcl-6 and BCoR transcripts after 1–2 h and downregulation by 4 h. While BCoR mRNA levels rapidly dropped to baseline within 4 hours, Bcl-6 mRNA showed a slower decline. With respect to the Bcl-6/BCoR transcript variants previously identified in leukocytes, a predominance of Bcl-6 mRNA variant 1 was observed in ECs. Comparably, endothelial BCoR transcripts mostly contained exons 5 and 8a throughout the induction phase (Figure 1B and Supplementary Figure S1).

### Endothelial Bcl-6 and BCoR expression is regulated by angiogenic stimuli

**Figure 2:**
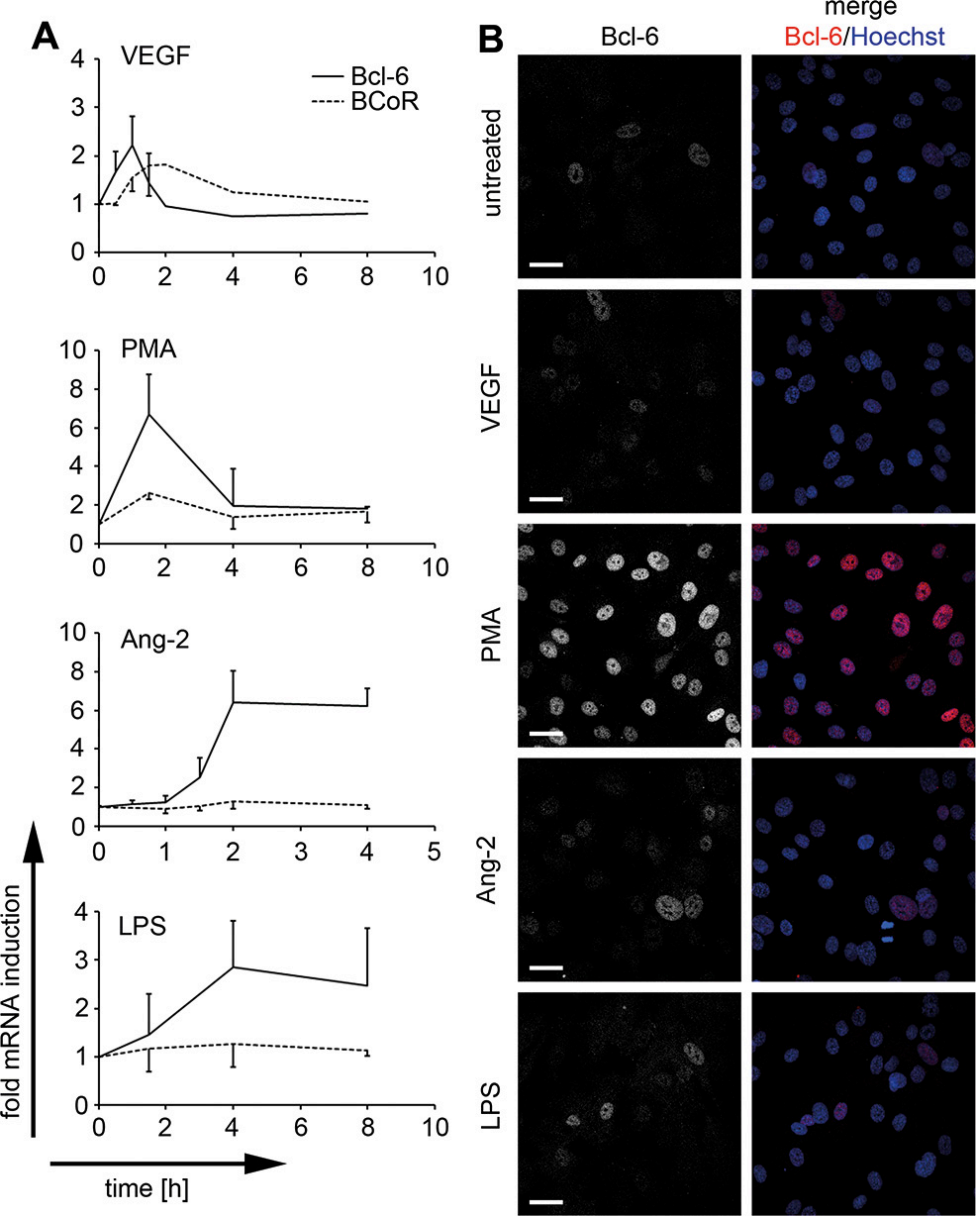
Endothelial Bcl-6/BCoR mRNA and protein induction by distinct stimuli BECs were treated with 30 ng/ml VEGF, 250 ng/ml Ang-2, 1 μg/ml LPS, 1 μg/ml PMA or were left untreated (*N* = 2). (**A**) Bcl-6/BCoR mRNA expression was measured by real-time PCR at the indicated time points. (**B**) After 3 h, cells were fixed and stained for confocal laser scanning microscopy with a fluorescence-labeled antibody against Bcl-6. Hoechst 33342 was applied to counterstain nuclear DNA (scale bar = 20 μm).

Considering the angiogenic properties of cancer cells and the recently reported pro-sprouting function of Bcl-6 family member BAZF, we further investigated whether endothelial expression of Bcl-6/BCoR was subject to regulation by angiogenic stimuli. While both transcripts were only moderately (2-fold) induced by VEGF, a more substantial increase (3- to 7-fold) in mRNA expression was triggered by PMA, a phorbol ester directly activating the intracellular protein kinase C pathway (Figure 2A). Distinct from BCoR, Bcl-6 was also prominently induced by angiopoietin-2 (Ang-2) and by pro-inflammatory stimuli such as LPS. Transcript regulation by VEGF and PMA was rapid and transient, generally reaching peak values between 1 and 2 h of stimulation, while Ang-2 and LPS triggered a prolonged increase in Bcl-6 mRNA levels.

Protein expression of Bcl-6 was confirmed by confocal laser scanning microscopy and showed exclusively nuclear localization. Bcl-6 protein was weakly detectable in untreated ECs, was moderately increased by VEGF, LPS or angiopoietin-2, and was strongly induced by PMA stimulation for 3 hours (Figure 2B). BCoR protein was consistently below detection levels in immunofluorescence or immunoblotting experiments and could only be shown by overexpression, resulting in prominent co-localization with Bcl-6 (data not shown). However, we have previously reported that overexpression of Bcl-6 or BCoR leads to the formation of nuclear aggregates [34]. Subsequent functional assays were therefore based on gene silencing rather than overexpression to reveal the properties of the endogenous proteins.

### Bcl-6/BCoR control the expression of cell cycle regulators to promote endothelial proliferation

**Figure 3:**
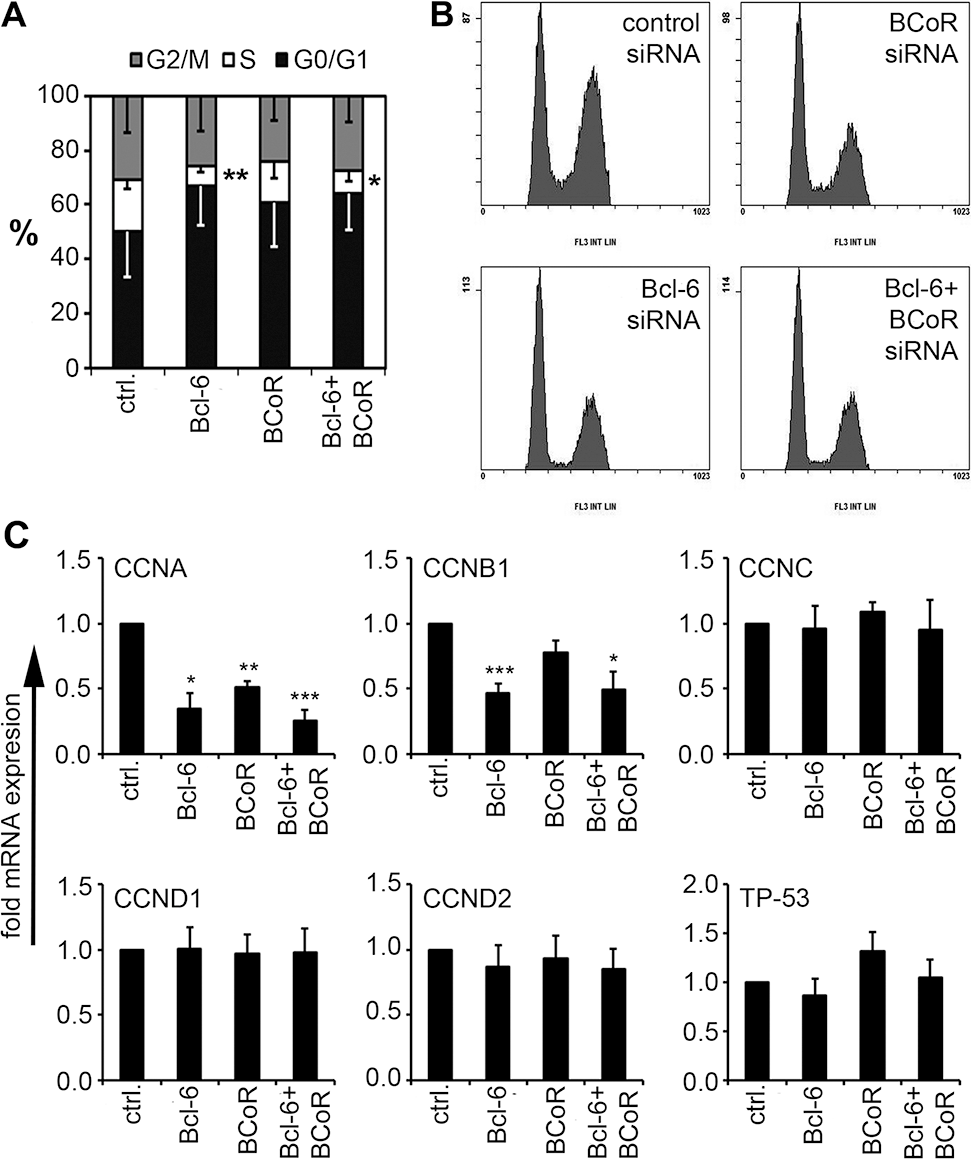
Impact of Bcl-6/BCoR silencing on endothelial cell cycle distribution (**A**, **B**) Cells were transfected with siRNA designed to silence the expression of Bcl-6 or BCoR. After 24 h, the cell cycle distribution was determined using propidium iodide staining. Flow cytometric data (B) were quantitated with MultiCycle software (A). (**C**) Cell extracts of transfected cells were further analyzed for mRNA expression of cell cycle regulators. Transcript levels were determined in relation to cells transfected with control siRNA. Mean values and standard deviations from 3 independent experiments are given. *T*-Test; *p < 0.05; **p < 0.01; ***p < 0.001.

As the expression of Bcl-6/BCoR was subject to regulation by angiogenic stimuli, their involvement in angiogenesis was further investigated, i.e., functional assays were conducted with human endothelial cells isolated from blood microvessels (BECs). The impact of Bcl-6/BCoR gene silencing (at 70% silencing efficiency, Supplementary Figure S2A) was evaluated with respect to angiogenic functions such as endothelial proliferation and migration. Silencing of Bcl-6 led to a significant reduction of BECs in S-phase (from 19 to 7%) indicating a G_0_/G_1_ cell cycle arrest (Figure 3A, 3B). BEC transfection with BCoR siRNA showed a comparable but weaker impact and no additive or synergistic effect of concomitant Bcl-6/BCoR silencing was observed. When transcripts of cell cycle regulators previously reported to be controlled by Bcl-6 in leukocytes were investigated, the downregulation of cyclin A and cyclin B1 corresponded with the observed effects (Figure 3C). In contrast, Bcl-6/BCoR gene silencing had no impact on the migratory behavior of BECs in transwell assays (Supplementary Figure S2B). Thus, Bcl-6/BCoR were found to positively affect endothelial proliferation and the cell cycle regulators cyclin A and cyclin B1.

### Silencing of Bcl-6/BCoR induces sprouting of endothelial cells *in vitro*

**Figure 4:**
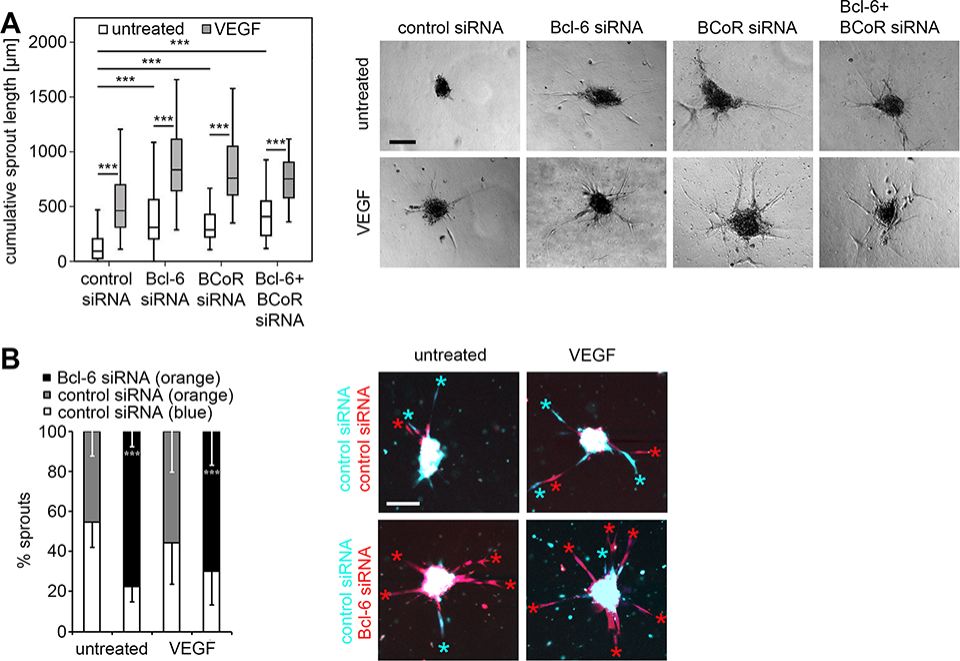
Impact of Bcl-6/BCoR silencing on endothelial sprouting Spheroids of BECs transfected with different siRNAs were embedded in a collagen matrix and treated with 30 ng/ml VEGF or were left untreated. After 18 h, the spheroids were fixed and the cumulative sprout length was determined per spheroid. A minimum of 10 randomly acquired spheroids was analyzed for each experiment. (**A**) Images of spheroids with prominent sprouting activity and boxplot illustration of statistical analysis from 6 independent sprouting experiments control siRNA: *N* = 100; control siRNA + VEGF: *N* = 90; Bcl-6 siRNA: *N* = 93; Bcl-6 siRNA + VEGF: *N* = 85; BCoR siRNA: *N* = 50; BCoR; siRNA + VEGF: *N* = 57; Bcl-6 + BCoR siRNA: *N* = 26; Bcl-6 + BCoR siRNA + VEGF: *N* = 45 spheroids). (**B**) BECs were transfected with Bcl-6 or control siRNA and labeled with an orange or blue cell tracker dye, respectively. Comparably, cells transfected with control siRNA were either labeled with a blue or orange cell tracker. Differently labeled cells were mixed at a ratio of 1:1, and generated spheroids were embedded in a collagen matrix with or without the addition of 30 ng/ml VEGF. After 18 h the spheroids were analyzed with an LSM780 confocal microscope for the prevalence (%) of orange or blue labeled sprouts as indicated by colored asterisks. Ten spheroids per condition were evaluated to quantitate sprout number and color and to conduct statistical analyses. Scale bars = 100 μm. *T*-Test (Bcl-6 siRNA versus control siRNA, orange label); *p < 0.05; **p < 0.01; ***p < 0.001.

When Bcl-6 and BCoR were silenced (either separately or simultaneously) BECs exhibited enhanced sprouting (Figure 4A). The cumulative sprout length per endothelial spheroid was determined after 18 hours and significantly increased from a median value of 90 μm for untreated cells (spontaneous sprouting) to 300 μm (single Bcl-6 or BCoR gene silencing) or 410 μm (combined gene silencing). Sprout induction by single or double gene knock-down did not differ significantly. Comparably, BEC stimulation with VEGF triggered a median cumulative sprout length of 460 μm. When Bcl-6/BCoR silencing was combined with VEGF treatment, sprout induction was further significantly enhanced to a maximum of 780 μm. Live cell imaging was applied to monitor sprouting over time and revealed a time course of sprout formation and elongation for Bcl-6/BCoR gene silencing comparable to VEGF stimulation (Supplementary Video S1). Negative controls were generally based on BECs transfected with control siRNA which were evaluated in addition to Bcl-6 and BCoR knock-down. To be able to evaluate the negative control in the same sample as the gene-silenced cells, BECs transfected with Bcl-6 siRNA or control siRNA were also labeled with distinct cell tracker dyes (orange or blue, respectively) and mixed at equal numbers in the sprouting assay (Figure 4B). The preferential formation of orange sprouts from Bcl-6 silenced cells (78%) was observed, thus supporting the conclusion that the endogenous Bcl-6/BCoR repressor complex is a negative regulator of endothelial sprout formation.

### Inhibition of Bcl-6 enhances angiogenic sprouting in the neonatal mouse retina

**Figure 5:**
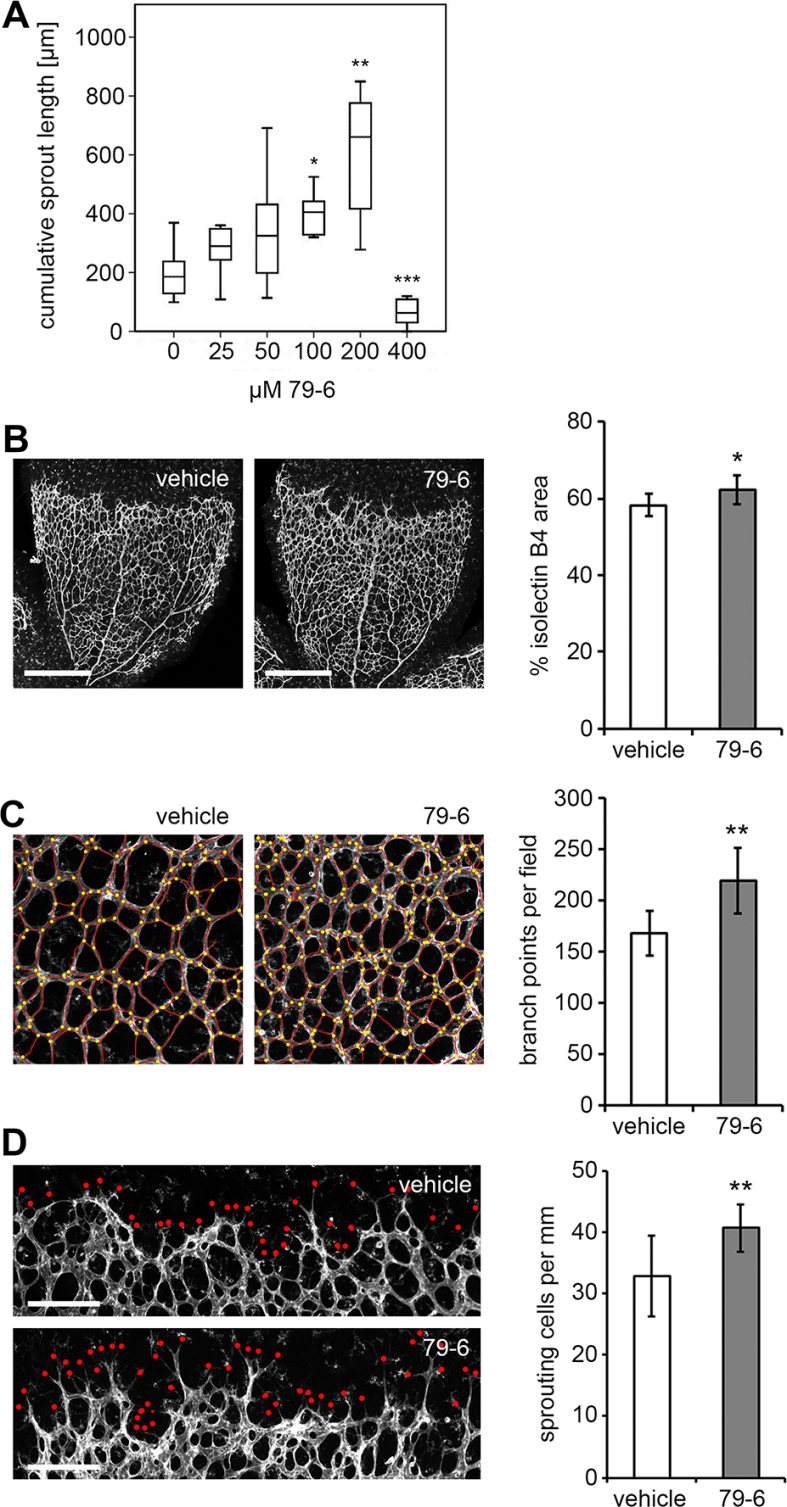
Impact of the Bcl-6 inhibitor 79-6 on capillary sprouting *in vitro* and *in vivo* (**A**) BEC spheroids were treated with increasing concentrations of the Bcl-6 inhibitor 79-6 for 18 h and the cumulative sprout length per spheroid was determined (*N* =10 spheroids for each condition). (**B**–**D**) C57Bl/6 wild type mice were injected intraperitoneally with 79-6 or vehicle on postnatal days P5 and P6. On P7, mice were sacrificed and retinas were prepared. Pictures show whole-mount isolectin B4 labeling of the retinal vasculature. (B) Confocal pictures of a quadrant vascular plexus as acquired with a Plan-Apochromat 5×/0.16 objective and tile-scanning mode. Scale bar = 500 μm. HistoQuest 3.5 software (TissueGnostics) served for the quantification of the isolectin B4 stained vessel area. Confocal images of the (C) vascular plexus and (D) angiogenic front as acquired with a Plan-Apochromat 10×/0.45 M27 objective. Scale bar = 200 μm. Images were further analyzed with Photoshop CS4 (Adobe Systems Inc.) for (C) quantification of branch points per microscopic field (as labeled by yellow dots), and (D) endothelial sprout tips (as labeled by red dots) per mm of the angiogenic front. *T*-Test (79-6 treatment *N* = 11, control *N* = 10); *p < 0.05; **p < 0.01.

Based on the *in vitro* assays, repression of Bcl-6 was expected to enhance angiogenesis in contrast to the previously reported knock-down of family member BAZF which resulted in the reduction of angiogenesis [29]. The mouse retina assay was applied to analyze the effect of Bcl-6 on angiogenesis *in vivo* using a soluble Bcl-6 inhibitor. The peptide inhibitor 79-6 has been developed to block Bcl-6 activity in non-Hodgkin lymphoma patients with aberrant Bcl-6 expression [33]. When tested in the *in vitro* BEC sprouting assay, 79-6 showed a dose-dependent induction of sprout formation up to 200 μM but was toxic at 400 μM (Figure 5A). Comparably, BEC proliferation was inhibited by 79-6 in a dose-dependent manner (Supplementary Figure S3). The optimized inhibitor concentration (200 μM) was also found to be effective in murine ECs. The compound was subsequently applied at a dose (50 μg/g) previously established in lymphoma models [33] to wild type C57BL/6 mice on postnatal days 5 und 6, and the mouse retinas were removed and stained for blood vessels on day 7. Bcl-6 expression was not monitored due to the low endogenous protein levels and the fact that inhibitor application would not be expected to alter protein levels. In contrast to the vehicle control, the application of 79-6 (Figure 5B–5D) significantly increased the number of tip cells (41 versus 33 tip cells per mm retinal front; *P* = 0.001), branch points (217 versus 168 branch points per microscopic field; *P* = 0.014), and vessel area (63 versus 59% isolectin B4^+^ area; *P* = 0.026). Thus, Bcl-6 was also confirmed to be an endogenous negative regulator of endothelial sprouting *in vivo*.

### Endothelial sprouting upon Bcl-6 silencing does not involve BAZF but is associated with changes in Notch target gene expression

**Figure 6:**
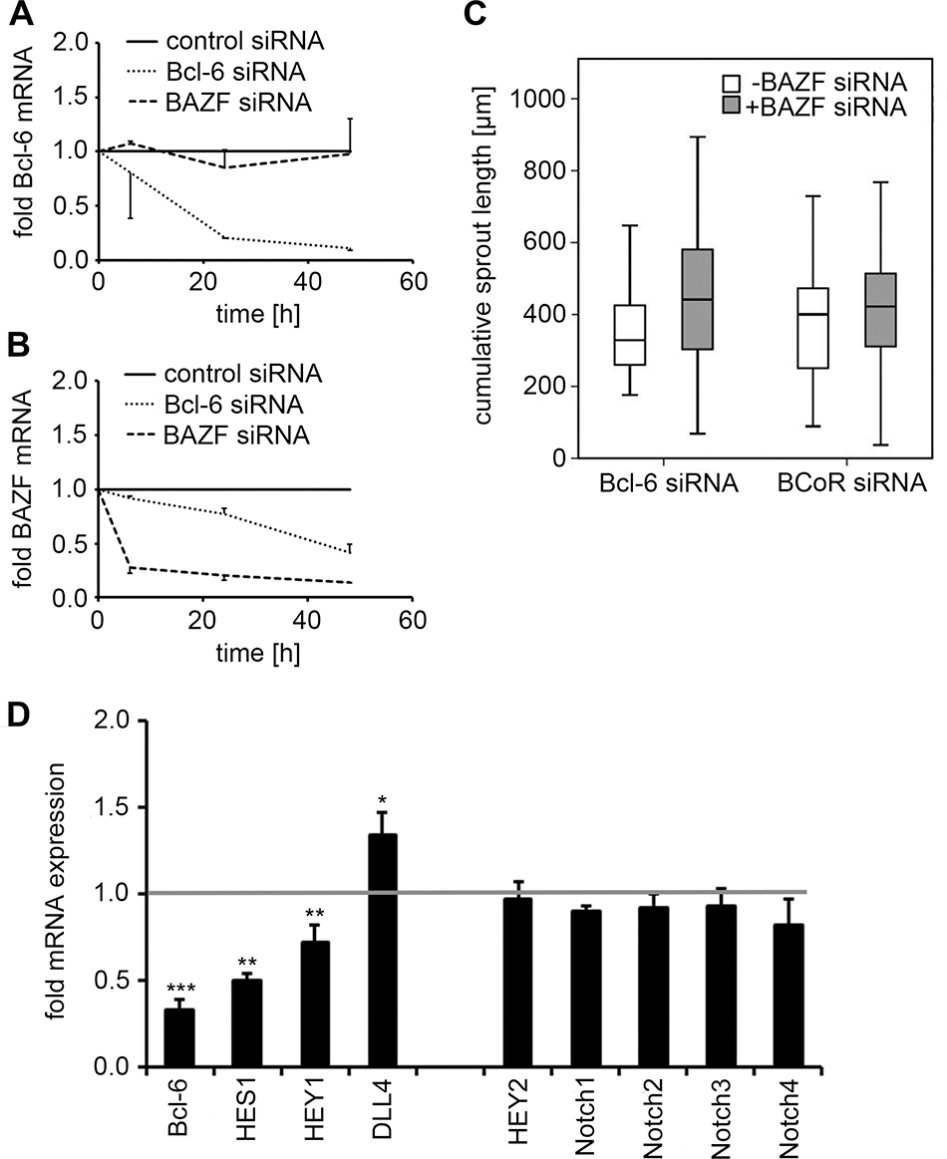
Analysis of BAZF and Notch targets following Bcl-6/BCoR silencing (**A**, **B**) BECs were transfected with control, Bcl-6 or BAZF siRNA. Cell extracts were prepared 8 to 48 h after transfection. mRNA expression levels of Bcl-6 (A) and BAZF (B) were determined by real-time PCR in relation to cells transfected with control siRNA. Mean values and standard deviations from 2 independent experiments are given. (**C**) Sprouting assay of BECs transfected with Bcl-6 or BCoR siRNA with or without BAZF siRNA. A minimum of 19 spheroids were evaluated per condition; the cumulative sprout length was compared (*T*-Test; n.s.). (**D**) BECs transfected with Bcl-6 siRNA were harvested 24 h after electroporation. Transcript levels for components of the Notch pathway were determined via real-time PCR in relation to control siRNA treated cells (value set to 1). Mean values and standard deviations from 2-3 independent experiments are shown: Bcl-6, HES1, HEY1, HEY2, DLL4: *N* = 3; Notch1-4: *N* = 2. *T*-Test; *p < 0.05; **p < 0.01; ***p < 0.001.

Since the Bcl-6 family member BAZF has been reported to promote angiogenic sprouting, a potential cross-talk between Bcl-6 and BAZF in terms of BAZF upregulation upon Bcl-6 silencing was investigated. When BECs were transfected with Bcl-6 siRNA, Bcl-6 transcript levels were reduced by 79–89% within 24-48 h. BAZF mRNA expression was affected by Bcl-6 silencing, but was decreased (rather than increased) by 58% at 48 h (Figure 6A, 6B). Conversely, BAZF siRNA was applied and showed an efficiency of BAZF silencing ranging at 79–86%. Knock-down of BAZF gene expression had no substantial effect on Bcl-6 transcript levels.

When a potential cross-talk of Bcl-6 and BAZF was tested at the functional level, the concomitant transfection of BECs with Bcl-6 (or BCoR) siRNA and BAZF siRNA did not prevent the induction of angiogenic sprouts (i.e. a median cumulative sprout length of 320–420 μm was triggered by Bcl-6/BCoR silencing in the absence or presence of BAZF siRNA; Figure 6C).

Since BAZF was reported to regulate Notch target gene expression [29], the impact of Bcl-6 silencing on components of the Notch pathway was investigated in more detail. Knock-down of Bcl-6 expression by 67% (at 24 h) was accompanied by a 50% reduction in HES1 and a 28% decrease in HEY1 transcript levels. Conversely, DLL4 mRNA expression was increased by 35% upon BEC transfection with Bcl-6 siRNA (Figure 6D). Of note, Bcl-6 silencing had no impact on transcript levels of HEY2 or Notch 1-4. BCoR silencing showed a comparable trend for HES1 and DLL4 regulation which was, however, not significant (data not shown). Thus, Bcl-6 (distinct from BAZF) was identified as a positive regulator of Notch target genes HES1 and HEY1 and a negative regulator of DLL4 expression, which are considered determinants of the stalk cell phenotype.

### Therapeutic inhibition of Bcl-6 in mouse cancer xenografts does not promote tumor growth despite a moderate tendency to increased microvessel density

**Figure 7:**
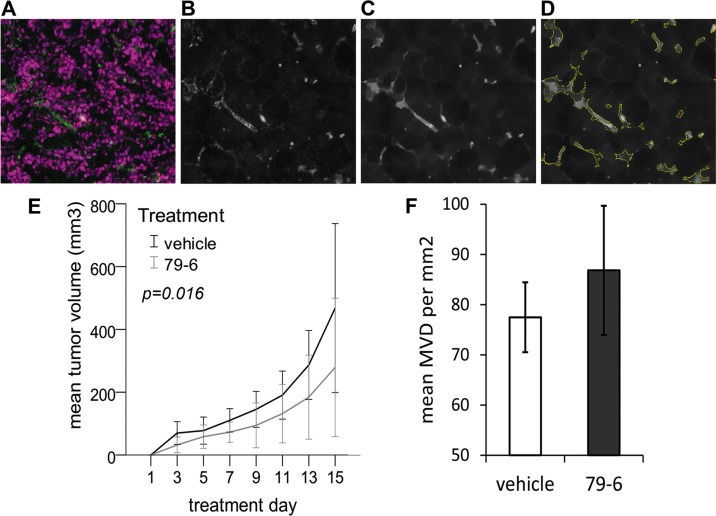
Effect of the Bcl-6 inhibitor 79-6 on HT-29 tumor growth and microvessel density BALB/c nude mice received subcutaneous injections of 1 × 10^7^ tumor cells into the right flank and were subsequently treated by intraperitoneal injections of 79-6 (*N* = 8) or vehicle (*N* = 8) for 14 consecutive days. Tumor growth (**E**) was recorded every second day. One day after the last treatment, animals were sacrificed and cryosections of tumors were stained for the endothelial marker CD31. Analysis of microvessel density (MVD) was based on automated tissue scanning by TissueFAXS and an algorithm developed with StrataQuest 5.0 software (TissueGnostics) as exemplified in **A**–**D**. Based on fluorescence images (A) of cell nuclei (purple) and endothelial CD31 expression (green), the FITC channel (CD31) was extracted to an 8-bit image, followed by a blurring algorithm (B) after which a morphological mask (C) was applied. (D) Microvessels were identified and quantitated based on kernel radius and background threshold settings which were manually adapted for each batch of scanned images. Results (mean and standard deviation) from three independent analyses are shown in (**F**).

The observation that the Bcl-6 inhibitor 79-6 enhanced endothelial sprouting *in vitro* and *in vivo* led to the pertinent question whether the anti-cancer compound might exert undesirable, tumor-promoting effects by boosting tumor angiogenesis. Hence, the effect of 79-6 therapy was evaluated in a mouse xenograft model with human HT-29 colorectal cancer cells (Figure 7). Tumor volume was recorded over two weeks of therapy, after which the tumor was excised and microvessel density was determined in cryosections stained for endothelial CD31 expression by automated tissue scanning, signal extraction and detection algorithm (Figure 7A–7D). However, tumor growth in mice treated with 79-6 was significantly reduced (*P* = 0.016) compared to control animals resulting in a mean tumor volume of 279 versus 468 mm^3^ at the end of the 14-day treatment period (Figure 7E). Despite the reduced growth, tumors of 79-6 treated animals showed a small tendency to a higher mean microvessel density, which was, however, not statistically significant (*P* = 0.330; Figure 7F). When a second xenograft model based on the Bcl-6 independent diffuse large B-cell lymphoma (DLBCL) Toledo cell line was tested, application of the 79-6 inhibitor comparably did not enhance tumor growth (data not shown).

## DISCUSSION

This study presents the first evidence for a functional role of Bcl-6 and BCoR in endothelial sprouting and proliferation. While Bcl-6 was previously detected in human umbilical vein endothelial cells and found to mediate the anti-inflammatory repression of adhesion molecules in response to the activation of peroxisome proliferator-activated receptor delta [32], BCoR has not been investigated in ECs to date. Thus, based on a gene expression array showing induction of BCoR mRNA in primary microvessel endothelial cells in response to tumor-derived stimuli, we conducted an investigation to characterize the expression and function of this transcriptional repressor complex in ECs in more detail. In contrast to the initial microarray analysis which did not detect regulation of Bcl-6 gene expression (presumably due to the sensitivity of the applied oligonucleotide probe set), the subsequent transcript analysis by real-time PCR involved several primer combinations and reproducibly revealed the regulation of both components, Bcl-6 and BCoR, by tumor-derived stimuli. To gain more information on the gene transcription in endothelial cells, splice variants previously reported for other cell types were investigated. Generation of the Bcl-6 V2 transcript was rare indicating that the majority of synthesized Bcl-6 mRNA was of the functional V1 type and expected to be translated [6, 7]. Since BCoR constitutes a large 200 kDa protein encoded by 15 exons [8], the exon structure was verified for endothelial BCoR mRNA by qualitative PCR (Supplementary Figure S1) and the known splice variants were subsequently quantified by real-time PCR (Figure 1). Of interest, more than 90% of BCoR transcripts contained exon 8a which encodes a 34 amino acid stretch required for BCoR interaction with the transcription factor AF9 [9]. Thus, exon usage would allow for alternative BCoR binding partners in ECs. However, the comparable phenotype of Bcl-6 and BCoR silencing in endothelial cell cycle and sprout formation suggests that the two proteins act in complex with respect to these functions. This notion is further supported by the observation that combined gene silencing had no additive effect.

The attempts to document co-localization of Bcl-6 and BCoR in endothelial nuclei were hampered by the detection limit for endogenous BCoR protein. As we have previously reported, transcript and protein levels of Bcl-6/BCoR are tightly controlled and rapidly downregulated upon transient transfection of endothelial cells with expression plasmids [34]. While the two proteins are found to co-localize in nuclear speckles within the first 6 hours post transfection, expression is promptly shut down thereafter (without cellular toxicity) pointing to a negative feedback mechanism as has previously been suggested for B-cells [35]. Cells that fail to downregulate Bcl6/BCoR expression exhibit the artificial aggregation of Bcl-6 and BCoR in large nuclear spheres which leads to remarkable structural changes of the nucleus [34]. In line, we have found that endogenous Bcl-6/BCoR protein levels are generally low and difficult to detect by immunofluorescence or immunoblotting. The induction of gene transcription by stimuli was transient and comparably showed rapid downregulation. Hence, the functional characterization of this transcriptional repressor complex was based on gene silencing rather than induction or overexpression and identified Bcl-6/BCoR as an endogenous repressor of endothelial sprouting.

Transfection of endothelial cells with Bcl-6 and/or BCoR siRNA had a profound effect, specifically, it triggered sprout formation in the absence of additional stimuli. Thus, our *in vitro* investigations support the notion that Bcl-6/BCoR constitute an endogenous transcription factor complex which prevents endothelial sprouting and tip cell formation. The additive effect of VEGF treatment and Bcl-6/BCoR silencing indicates that activation of sprouting by VEGF can be further enhanced by removing the Bcl-6/BCoR “brake” in BECs. The observed changes in Notch target gene expression (HES1, HEY1 and DLL4) upon Bcl-6 silencing suggest that the transcription factor promotes a stalk cell phenotype. This is in line with the sustained and strong induction of Bcl-6 mRNA by Ang-2, while VEGF showed only weak effects on Bcl-6/BCoR expression. Comparably, Bcl-6 silencing also led to a 50% reduction in Tie2 receptor expression of endothelial cells (*P* = 0.054; data not shown) which further indicates a link between Bcl-6 expression and Ang-2/Tie2 signaling, a hallmark of stalk cells [36]. Furthermore, stalk cells are required to proliferate to elongate the sprout [37], a process which we also found to be supported by Bcl-6/BCoR action. Loss of Bcl-6/BCoR expression was accompanied by a significant reduction in cyclin A and cyclin B1 transcripts and a reduced transition of cells into S-phase. A positive correlation between Bcl-6 and cyclin A or cyclin B1 expression has previously been reported for B-cell lymphomas where Bcl-6 promotes cell proliferation [24, 38]. Of note, the majority of transcripts found to be regulated upon Bcl6/BCoR silencing showed a positive correlation with Bcl-6/BCoR expression (i.e. were reduced in endothelial cells transfected with Bcl-6/BCoR siRNA). Considering that Bcl-6/BCoR constitute a transcriptional repressor complex, this positive association may seem counterintuitive. However, indirect modes of action by repressing or sequestering other inhibitors to effectively promote the expression of target genes such as c-myc and cyclin B1 have previously been reported for Bcl-6 [24]. A comparable, more complex mechanism of Bcl-6/BCoR action in endothelial cells involving downstream pathways of indirect target gene regulation is therefore likely and will require extended further investigation.

Remarkably, Bcl-6 was found to play an opposite role in endothelial sprouting as compared to its family member BAZF which promotes the tip cell phenotype by blocking Notch signaling and reducing Notch target gene expression [29]. Due to the fact that Bcl-6 and BAZF may form heterodimers [28], a potential mechanism of BAZF “liberation” upon Bcl-6 knock-down could be envisioned to result in sprout formation. However, when both genes were silenced concomitantly, endothelial sprouting did occur which demonstrated that BAZF was not involved in the sprout induction upon Bcl-6 knock-down. Nevertheless, Notch target gene expression was altered by Bcl-6 silencing and indicated a cross-talk of Bcl-6 and Notch signaling in reinforcing the stalk cell phenotype. A negative cross-talk between Bcl-6/BCoR and Notch signaling has previously been described in the communication between neighboring cells during developmental left-right patterning. In this case, the Bcl-6/BCoR repressor complex was found to bind to and inhibit individual Notch target genes [39].

The *in vitro* investigations on endothelial sprouting triggered by Bcl-6/BCoR gene knock-down were further confirmed by an *in vivo* model of physiological angiogenesis. The commercially obtainable Bcl-6 inhibitor 79-6 was applied in the neonatal retina angiogenesis assay since an endothelial specific gene knock-out mouse was not available and Bcl-6 deficient mice suffer from severe immunological and inflammatory defects [16]. Tip cell formation was significantly increased when Bcl-6 was inhibited systemically during postnatal days 5 and 6, thus confirming that Bcl-6 constitutes an endogenous inhibitor of endothelial sprouting. The fact that 79-6 seemed less potent in *in vivo* angiogenesis as compared to *in vitro* sprouting assays may relate to the stability and delivery of the compound in circulation or might be explained by additional (so far unknown) effects on other cell types involved in *in vivo* angiogenesis.

While the mouse retina model investigated physiological angiogenesis, a possibly promoting effect of Bcl-6 inhibition on pathological tumor vascularization was of particular concern. Since Bcl-6 is the most frequently involved oncogene in diffuse large B cell lymphoma, the Bcl-6 inhibitor 79-6 was developed to suppress lymphoma cell growth [33]. A potentially adverse effect of Bcl-6 blockade in cancer therapy would be feasible considering the important role of angiogenesis in tumor expansion (or bone marrow angiogenesis in lymphoproliferative disease). However, when we tested 79-6 in mouse xenograft models of DLBCL or colorectal cancer, tumor growth was not enhanced. In contrast, growth of colorectal carcinomas was reduced despite a small tendency to increased microvessel density. Since 79-6 was administered systemically, the net impact on tumor growth was likely the result of combined inhibitor effects on cancer cells, tumor stroma and infiltrating leukocytes which decreased rather than increased tumor expansion. Furthermore, it is essential to note that endothelial Bcl-6/BCoR were found to be induced by tumor-derived stimuli in our initial experiments. Cancer cells secrete a variety of factors such as VEGF or Ang-2 which affect the balance required to positively and negatively control tip/stalk cell communication during the angiogenic process. In this pathological setting, it is conceivable that Bcl-6 blockade by 79-6 could not further enhance tumor-driven angiogenesis. With respect to the clinical application of Bcl-6 inhibitors, these findings may thus provide valuable novel information.

## MATERIALS AND METHODS

### Cell culture

Human microvessel endothelial cells were isolated from foreskin via anti-CD31 antibody coupled Dynabeads (Invitrogen, Thermo Fisher Scientific Inc., Waltham, MA). BECs were further separated from lymphatic endothelial cells by anti-podoplanin Dynabeads. ECs were grown to confluence and incubated with tumor-conditioned medium from HT-29, LS-174T and SW620 colorectal carcinoma cultures for the indicated time periods. When defined stimuli were tested, ECs were exposed to VEGF-A at 30 ng/ml, LPS at 1 μg/ml, PMA at 100 ng/ml or Ang-2 at 250 ng/ml.

### Transfection of ECs with siRNA

The following siRNA solutions at 20 μM (Invitrogen) were added to 2×10^6^ ECs in 400 μl RPMI1640 medium containing 10% FBS and cells were electroporated in a 4 mm cuvette at 200 V and 1200 μF with a Gene Pulser Xcell system (Bio-Rad Laboratories Inc., Hercules, CA): Bcl-6 HSS100966 (30 μl), Bcl-6 HSS100968 (30 μl), BCoR HSS123439 (20 μl), BAZF HSS137953 (20 μl); negative control siRNA with low GC content (matching volume).

### Assessment of gene transcripts

Standard procedures for total RNA isolation, cDNA generation and qualitative or quantitative real-time PCR were applied [34]. Primer sequences and PCR conditions are specified in the detailed Supplementary Methods and Supplementary Tables S2–S4.

### Immunocytochemistry

Cells on glass slides were permeabilized and stained with Bcl-6 antibody M7211 (Dako Glostrup, Denmark) at 2 μg/ml. AlexaFluor555-conjugated secondary antibody was combined with Hoechst 33342 for nuclear staining (Invitrogen). Mounted samples were examined with a Zeiss LSM780 confocal microscope (Carl Zeiss AG, Oberkochen, Germany).

### Cell cycle analysis

24 h after transfection, BECs were permeabilized and treated with 500 μg/ml RNase H and 50 μg/ml propidium iodide. Cell cycle distribution was analyzed with a Gallios Flow Cytometer (Beckman Coulter, Fullerton, CA) and Multicycle^TM^ AV Software (Phoenix Flow Systems, San Diego, CA).

### Sprouting assay

Transfected BECs were left unstained or were labeled with CellTracker^TM^ Blue CMF_2_HC or Orange CMTMR (Thermo Fisher). BEC spheroids were generated in methyl cellulose over-night and seeded in methyl cellulose mixed with rat tail collagen (ratio 1:1) and 10% FBS. Where indicated, VEGF was added at 30 ng/ml. Sprout formation was evaluated after 18 h using an Axiovert 40 CFL microscope (Carl Zeiss AG) or by live cell imaging with a Cell-IQ^®^ MLF system (CM Technologies, Tampere, Finland).

### Mouse retina angiogenesis model

All animal experiments were approved by the local ethics committee (BMWFW-66.009/0155-WF/V/3b/2015). C57Bl/6 wild type littermates were injected at postnatal days P5 and P6 with 50 μg/g 79-6 (Calbiochem – Merck, Darmstadt, Germany) in 100 μl vehicle (peanut oil + 10% ethanol) or vehicle only. Retinas were isolated at P7 and stained with biotinylated isolectin B4 (Vector Laboratories, Burlingame, CA) and streptavidin-AlexaFluor555 conjugate (Invitrogen). Whole-mount retinas were analyzed with a Zeiss LSM780 confocal microscope, Photoshop CS4 and HistoQuest 3.5 software (TissueGnostics, Vienna, Austria).

### Mouse xenograft model of colorectal cancer

A total of 1×10^7^ HT-29 cells were subcutaneously injected into the right flank of BALB/c/Him nude mice (Division for Laboratory Animal Science and Genetics, Medical University of Vienna, Himberg, Austria). On the following day, animals were randomized into two groups of 8 mice which received a daily dose of 50 μg Bcl-6 inhibitor 79-6 (or vehicle) per gram mouse weight by intraperitoneal injection for 14 consecutive days. Tumor growth was recorded every second day and tumors were excised one day after the last treatment. Cryosections were incubated with primary antibody #550274 directed against the endothelial marker CD31 (Becton Dickinson, Franklin Lakes, NJ) at 1:50 dilution for 60 min, followed by treatment with donkey anti-rat AlexaFluor488 at 1:800 and TOTO-3 DNA stain (Thermo Fisher) at 1:500 dilution for 30 min. Images were acquired with TissueFAXS, an automated multi-channel immunofluorescence detection system (TissueGnostics, Vienna, Austria) and microvessel density was determined using an algorithm developed with StrataQuest 5.0 software (TissueGnostics) as specified in the detailed Supplementary Methods. A minimum of three distinct regions within each tumor (in tissue sections at 30–50 μm distance) was evaluated, with an average scanned area of 58 mm^2^ per tumor.

## SUPPLEMENTARY MATERIALS







## References

[1] Baron BW, Stanger RR, Hume E, Sadhu A, Mick R, Kerckaert JP, Deweindt C, Bastard C, Nucifora G, Zeleznik-Le N, McKeithan TW (1995). BCL6 encodes a sequence-specific DNA-binding protein. Genes Chromosomes Cancer.

[2] Kawamata N, Miki T, Ohashi K, Suzuki K, Fukuda T, Hirosawa S, Aoki N (1994). Recognition DNA sequence of a novel putative transcription factor, BCL6. Biochem Biophys Res Commun.

[3] Lemercier C, Brocard MP, Puvion-Dutilleul F, Kao HY, Albagli O, Khochbin S (2002). Class II histone deacetylases are directly recruited by BCL6 transcriptional repressor. J Biol Chem.

[4] Dhordain P, Albagli O, Lin RJ, Ansieau S, Quief S, Leutz A, Kerckaert JP, Evans RM, Leprince D (1997). Corepressor SMRT binds the BTB/POZ repressing domain of the LAZ3/BCL6 oncoprotein. Proc Natl Acad Sci USA.

[5] Huynh KD, Bardwell VJ (1998). The BCL-6 POZ domain and other POZ domains interact with the co-repressors N-CoR, SMRT. Oncogene.

[6] Pantano S, Jarrossay D, Saccani S, Bosisio D, Natoli G (2006). Plastic downregulation of the transcriptional repressor BCL6 during maturation of human dendritic cells. Exp Cell Res.

[7] Tsuyama N, Danjoh I, Otsuyama K, Obata M, Tahara H, Ohta T, Ishikawa H (2005). IL-6-induced Bcl6 variant 2 supports IL-6-dependent myeloma cell proliferation and survival through STAT3. Biochem Biophys Res Commun.

[8] Huynh KD, Fischle W, Verdin E, Bardwell VJ (2000). BCoR, a novel corepressor involved in BCL-6 repression. Genes Dev.

[9] Srinivasan RS, de Erkenez AC, Hemenway CS (2003). The mixed lineage leukemia fusion partner AF9 binds specific isoforms of the BCL-6 corepressor. Oncogene.

[10] Horn D, Chyrek M, Kleier S, Luttgen S, Bolz H, Hinkel GK, Korenke GC, Riess A, Schell-Apacik C, Tinschert S, Wieczorek D, Gillessen-Kaesbach G, Kutsche K (2005). Novel mutations in BCOR in three patients with oculo-facio-cardio-dental syndrome, but none in Lenz microphthalmia syndrome. Eur J Hum Genet.

[11] Hilton EN, Manson FD, Urquhart JE, Johnston JJ, Slavotinek AM, Hedera P, Stattin EL, Nordgren A, Biesecker LG, Black GC (2007). Left-sided embryonic expression of the BCL-6 corepressor, BCOR, is required for vertebrate laterality determination. Hum Mol Genet.

[12] Wamstad JA, Bardwell VJ (2007). Characterization of Bcor expression in mouse development. Gene Expr Patterns.

[13] Wamstad JA, Corcoran CM, Keating AM, Bardwell VJ (2008). Role of the transcriptional corepressor Bcor in embryonic stem cell differentiation and early embryonic development. PLoS One.

[14] Cattoretti G, Pasqualucci L, Ballon G, Tam W, Nandula SV, Shen Q, Mo T, Murty VV, Dalla-Favera R (2005). Deregulated BCL6 expression recapitulates the pathogenesis of human diffuse large B cell lymphomas in mice. Cancer Cell.

[15] Jardin F, Sahota SS (2005). Targeted somatic mutation of the BCL6 proto-oncogene and its impact on lymphomagenesis. Hematology.

[16] Dent AL, Shaffer AL, Yu X, Allman D, Staudt LM (1997). Control of inflammation, cytokine expression, and germinal center formation by BCL-6. Science.

[17] Ye BH, Cattoretti G, Shen Q, Zhang J, Hawe N, de Waard R, Leung C, Nouri-Shirazi M, Orazi A, Chaganti RS, Rothman P, Stall AM, Pandolfi PP (1997). The BCL-6 proto-oncogene controls germinal-centre formation and Th2-type inflammation. Nat Genet.

[18] Toney LM, Cattoretti G, Graf JA, Merghoub T, Pandolfi PP, Dalla-Favera R, Ye BH, Dent AL (2000). BCL-6 regulates chemokine gene transcription in macrophages. Nat Immunol.

[19] Albagli O, Lantoine D, Quief S, Quignon F, Englert C, Kerckaert JP, Montarras D, Pinset C, Lindon C (1999). Overexpressed BCL6 (LAZ3) oncoprotein triggers apoptosis, delays S phase progression and associates with replication foci. Oncogene.

[20] Albagli O, Lindon C, Lantoine D, Quief S, Puvion E, Pinset C, Puvion-Dutilleul F (2000). DNA replication progresses on the periphery of nuclear aggregates formed by the BCL6 transcription factor. Mol Cell Biol.

[21] Shvarts A, Brummelkamp TR, Scheeren F, Koh E, Daley GQ, Spits H, Bernards R (2002). A senescence rescue screen identifies BCL6 as an inhibitor of anti-proliferative p19(ARF)-p53 signaling. Genes Dev.

[22] Phan RT, Saito M, Basso K, Niu H, Dalla-Favera R (2005). BCL6 interacts with the transcription factor Miz-1 to suppress the cyclin-dependent kinase inhibitor p21 and cell cycle arrest in germinal center B cells. Nat Immunol.

[23] Kusam S, Vasanwala FH, Dent AL (2004). Transcriptional repressor BCL-6 immortalizes germinal center-like B cells in the absence of p53 function. Oncogene.

[24] Shaffer AL, Yu X, He Y, Boldrick J, Chan EP, Staudt LM (2000). BCL-6 represses genes that function in lymphocyte differentiation, inflammation, and cell cycle control. Immunity.

[25] Hosokawa Y, Maeda Y, Seto M (2001). Target genes downregulated by the BCL-6/LAZ3 oncoprotein in mouse Ba/F3 cells. Biochem Biophys Res Commun.

[26] Okabe S, Fukuda T, Ishibashi K, Kojima S, Okada S, Hatano M, Ebara M, Saisho H, Tokuhisa T (1998). BAZF, a novel Bcl6 homolog, functions as a transcriptional repressor. Mol Cell Biol.

[27] Sakashita C, Fukuda T, Okabe S, Kobayashi H, Hirosawa S, Tokuhisa T, Miyasaka N, Miura O, Miki T (2002). Cloning and characterization of the human BAZF gene, a homologue of the BCL6 oncogene. Biochem Biophys Res Commun.

[28] Takenaga M, Hatano M, Takamori M, Yamashita Y, Okada S, Kuroda Y, Tokuhisa T (2003). Bcl6-dependent transcriptional repression by BAZF. Biochem Biophys Res Commun.

[29] Ohnuki H, Inoue H, Takemori N, Nakayama H, Sakaue T, Fukuda S, Miwa D, Nishiwaki E, Hatano M, Tokuhisa T, Endo Y, Nose M, Higashiyama S (2012). BAZF, a novel component of cullin3-based E3 ligase complex, mediates VEGFR, Notch cross-signaling in angiogenesis. Blood.

[30] Ribatti D, Crivellato E (2012). “Sprouting angiogenesis”, a reappraisal. Dev Biol.

[31] Miwa D, Sakaue T, Inoue H, Takemori N, Kurokawa M, Fukuda S, Omi K, Goishi K, Higashiyama S (2013). Protein kinase D2 and heat shock protein 90 beta are required for BCL6-associated zinc finger protein mRNA stabilization induced by vascular endothelial growth factor-A. Angiogenesis.

[32] Fan Y, Wang Y, Tang Z, Zhang H, Qin X, Zhu Y, Guan Y, Wang X, Staels B, Chien S, Wang N (2008). Suppression of pro-inflammatory adhesion molecules by PPAR-delta in human vascular endothelial cells. Arterioscler Thromb Vasc Biol.

[33] Cerchietti LC, Ghetu AF, Zhu X, Da Silva GF, Zhong S, Matthews M, Bunting KL, Polo JM, Fares C, Arrowsmith CH, Yang SN, Garcia M, Coop A (2010). A small-molecule inhibitor of BCL6 kills DLBCL cells *in vitro* and *in vivo*. Cancer Cell.

[34] Buchberger E, El Harchi M, Payrhuber D, Zommer A, Schauer D, Simonitsch-Klupp I, Bilban M, Brostjan C (2013). Overexpression of the transcriptional repressor complex BCL-6/BCoR leads to nuclear aggregates distinct from classical aggresomes. PLoS One.

[35] Mendez LM, Polo JM, Yu JJ, Krupski M, Ding BB, Melnick A, Ye BH (2008). CtBP is an essential corepressor for BCL6 autoregulation. Mol Cell Biol.

[36] del Toro R, Prahst C, Mathivet T, Siegfried G, Kaminker JS, Larrivee B, Breant C, Duarte A, Takakura N, Fukamizu A, Penninger J, Eichmann A (2010). Identification and functional analysis of endothelial tip cell-enriched genes. Blood.

[37] Gerhardt H, Golding M, Fruttiger M, Ruhrberg C, Lundkvist A, Abramsson A, Jeltsch M, Mitchell C, Alitalo K, Shima D, Betsholtz C (2003). VEGF guides angiogenic sprouting utilizing endothelial tip cell filopodia. J Cell Biol.

[38] Bai M, Agnantis NJ, Skyrlas A, Tsanou E, Kamina S, Galani V, Kanavaros P (2003). Increased expression of the bcl6 and CD10 proteins is associated with increased apoptosis and proliferation in diffuse large B-cell lymphomas. Mod Pathol.

[39] Sakano D, Kato A, Parikh N, McKnight K, Terry D, Stefanovic B, Kato Y (2010). BCL6 canalizes Notch-dependent transcription, excluding Mastermind-like1 from selected target genes during left-right patterning. Dev Cell.

