# Supplementary material for: Inhibition of the transcriptional repressor complex Bcl-6/BCoR induces endothelial sprouting but does not promote tumor growth
Source: Oncotarget. 2016 Nov 21;8(1):552–64. doi: 10.18632/oncotarget.13477 (PMC5352177; doi:10.18632/oncotarget.13477)
Supplement: Supplementary file 1 [file oncotarget-08-552-s001.pdf]

Inhibition of the transcriptional repressor complex Bcl-6/BCoR induces endothelial sprouting but does not promote tumor growth

Supplementary Materials

SUPPLEMENTARY METHODS

Cell culture

Primary human microvessel endothelial cells (ECs) as isolated from human foreskin by dispase digest were purified to > 98% purity via anti-CD31 antibody coupled Dynabeads (Invitrogen, Thermo Fisher Scientific Inc., Waltham, MA). BECs (blood endothelial cells) were further separated from lymphatic endothelial cells by negative selection with the use of anti-podoplanin antibody coupled Dynabeads. ECs were cultured in gelatin-coated dishes containing EGM2-MV growth medium (Lonza, Walkersville, MD) without VEGF supplementation in a 5% CO_2 atmosphere. All endothelial isolates were characterized by flow cytometry for EC markers (i.e. for CD31, CD34 and podoplanin expression, and for E-selectin induction following stimulation with 100 ng/ml TNFα for 4 h). Human colon carcinoma cell lines HT-29 (HTB-38), LS-174T (CL-188) and SW620 (CLL-227) were originally obtained from ATCC (in 5/2000), frozen in aliquots and used in experiments below passage 25 after delivery. These cell lines were cultured in McCoy5A, EMEM or Leibowitz L15 growth medium, respectively. Media were supplemented with 10% FBS (fetal bovine serum), 2 mM L-glutamine, 100 µg/ml streptomycin and 100 U/ml penicillin. Sodium pyruvate at 1 mM was added to EMEM. For stimulation with tumor cell supernatant, ECs were seeded in 6-well dishes and grown to confluence within 24 h. Medium was changed to EGM2-MV without supplemental growth factors 24 h prior to stimulation. Simultaneously, tumor cells were prepared by plating the cells in EGM2-MV without growth factors in 6-well dishes at the following cell densities: HT-29 and SW620 at 4×10^6 cells, LS174T at 8×10^6 cells. The following day, fresh medium was supplied to tumor cell cultures, supernatants were collected after 24 h and centrifuged to remove cell remnants. Confluent EC cultures in 6-wells were then incubated with tumor-conditioned medium for the indicated time periods. When defined stimuli were tested, ECs were seeded in EGM2-MV without growth factors as outlined above. VEGF-A at 30 ng/ml, LPS at 1 µg/ml, PMA at 100 ng/ml or angiopoietin-2 at 250 ng/ml were added for 30 min to 8 h before harvesting cells for RNA or protein analysis.

Microarray analysis

ECs (3×10^5) were seeded in complete EGM2-MV growth medium on polyethylene terephthalate transwells of 1 µm pore size (Becton Dickinson, Franklin Lakes, NJ, USA) to form a confluent layer within 24 h and were then cultured for another 24 h in EGM2-MV medium including 5% FBS but no additional growth factor supplements. HT-29 colon carcinoma cells were seeded in the same growth medium at 4×10^6 per 6-well and conditioned supernatant was collected from 24 to 48 h after cell seeding. EC cultures in transwells were left untreated or were stimulated by tumor-conditioned medium from the basal (experiment 1) or apical (experiment 2) side for 4 h prior to isolation of RNA (Qiagen, Venlo, Netherlands). 5 µg of total RNA were used to generate double-stranded cDNA by reverse transcription with a cDNA synthesis kit (Superscript Choice System; Life Technologies, Thermo Fisher Scientific Inc.). Labeled cRNA was prepared from the double-stranded cDNA by *in vitro* transcription using T7 RNA polymerase in the presence of biotin-11-CTP and biotin-16-UTP (Enzo, Farmington, NY). 15 µg of cRNA were fragmented and applied in the hybridization cocktail for HU133A arrays (Affymetrix, Santa Clara, CA) at 45°C for 16 h in a rotisserie oven at 60 rpm. The arrays were washed using a non-stringent buffer at 25°C, followed by a stringent buffer at 50°C. Signal intensity was calculated based on MAS5.0 normalized data which are accessible via GEO database (accession number GSE67914).

Transfection of ECs with siRNA

Cell transfection was carried out by electroporation. Cells were grown to 70–80% confluence, harvested and suspended in RPMI1640 medium (PAA Corp., Pasching, Austria) containing 10% FBS to obtain a cell count of 2×10^6 in 400 µl. siRNAs were added and cells were

subsequently electroporated in a 4 mm cuvette at 200 V and 1200 µF with a Gene Pulser Xcell system (Bio-Rad Laboratories Inc., Hercules, CA). Cells were analyzed or applied in assays 6 to 48 h after transfection. The following siRNAs (Invitrogen) were used: Bcl-6 HSS100966 (30 µl), Bcl-6 HSS100968 (30 µl), BCoR HSS123439 (20 µl), BAZF HSS137953 (20 µl). A Stealth Select siRNA with low GC content (Invitrogen) was applied as a negative control at comparable amounts. All siRNAs were provided at a concentration of 20 µM.

Quantitative real-time PCR

Total RNA was isolated from EC cultures with E.Z.N.A. MicroElute Total RNA Kit (Omega Bio-Tek Inc., Norcross, GA) according to manufacturer´s instructions. 500 ng RNA were reverse transcribed with oligo(dT) primers using the Finnzymes cDNA kit (Thermo Fisher). The generated cDNA was diluted 1:20 before PCR analysis. Real-time PCR was performed with either ABI SYBR Fast (Applied Biosystems, Foster City, CA) or qPCR MasterMix Plus Low ROX (Eurogentec, Searing, Belgium). Oligonucleotide primers for SYBR Green detection were used at 300 nM forward primer and 300 nM reverse primer. For TaqMan assays 450 nM forward primer, 450 nM reverse primer and 200 nM probe were applied. All primer sets spanned at least one exon/intron boundary and are listed in Supplementary Tables S2 and S3. Each sample was assayed in duplicate with the 7500 Fast PCR Detection System (Applied Biosystems) for 45 cycles of 15 sec at 95°C followed by 1 min at 60°C (standard qPCR) or 45 cycles of 3 sec at 95°C followed by 30 sec at 60°C (fast qPCR). Denaturing curves were established for all SYBR Green reactions to verify homogeneity of the amplified products. Transcript levels for target genes were calculated using the $E^{-\Delta\Delta Ct}$-method. The efficiency of reactions was calculated according to the equation $E = 10^{1/slope}$. β-actin and 36B4 were used as housekeeping genes.

Qualitative PCR

Exon structure and splice variants of BCoR were also characterized by qualitative PCR. Primers were positioned to cover entire exons (i.e. were placed in adjacent exons close to intron junctions resulting in PCR products in the range of 200 bp to 3 kb; Supplementary Table S4). For qualitative PCR, 2 µl cDNA was added to a 50 µl sample containing PCR buffer, 1.5 mM $MgCl_2$, (Invitrogen), 200 µM dNTPs (Amersham Biosciences Corp. Piscataway, NJ), 200 nM oligonucleotide primers and 1 U Taq polymerase (Invitrogen). The PCR was performed in a Biometra T3 Thermocycler (Biometra, Goettingen, Germany) with the following cycle conditions for exons 2, 3, 4, 4-6, 4s, 6/7, 8a/b and 9: 3 min at 94°C, 35 cycles of 1 min at 94°C, 1 min at 50°C, 1 min at 72°C followed by 10 min at 72°C. For exon 4, an 8 min extension time was included due to the exon length of 2.8 kb. Exons 10 to 14 were amplified as follows: 3 min at 94°C, 35 cycles of 45 sec at 94°C, 30 sec at 50°C, 1 min at 72°C followed by 10 min at 72°C. PCR products were subsequently analyzed on 1% agarose gels with ethidium bromide staining.

Immunocytochemistry and confocal microscopy

Cells were seeded on gelatin-coated glass cover slips. After 24 h in culture, cells were treated with stimuli (as described above) for 3 h, then fixed in 4% paraformaldehyde (PFA) for 10 min followed by permeabilization with 0.5% Triton X-100 for 7 min. The cells were washed extensively in PBS and blocked with 2% BSA for 20 min. Bcl-6 antibody M7211 (Dako Glostrup, Denmark) was added at 2 µg/ml for 1 h. Cells were again washed with PBS and incubated for 1 h with AlexaFluor555 conjugated secondary antibody (Invitrogen) at a 1:1000 dilution of a 2 mg/ml stock. For nuclear staining, 10 µg/ml Hoechst 33342 (Invitrogen) was added. After a final washing step the cover slips were dried and mounted onto glass slides with Fluoromount-G™ (SouthernBiotech, Birmingham, AL) anti-fade reagent. Stained samples were examined with a Zeiss LSM780 confocal microscope equipped with a 40×/1.4 oil DIC objective. Images were obtained and analyzed with Zen2010 software (Carl Zeiss AG, Oberkochen, Germany) and were further processed with Photoshop CS4 (Adobe Systems Inc., San Jose, CA).

Cell cycle analysis

BECs transfected with siRNA were seeded at subconfluent conditions and incubated for 24 hours. The cells were harvested and pellets were processed by drop-wise addition of 1 ml chilled 70% ethanol and careful mixing after each drop. The suspension was kept on ice for 30 min and collected by centrifugation. 1 ml of PBS containing 500 µg/ml RNase H and 50 µg/ml propidium iodide was added and the sample was incubated for 15 min at 37°C. The cell cycle distribution was analyzed by flow cytometry with a Gallios Flow Cytometer (Beckman Coulter, Fullerton, CA). Propidium iodide stained, single cells were selected by means of dual multiparameter analysis of peak height and integral fluorescence. Cell cycle distributions were deconvoluted using the Multicycle™ AV Software (Phoenix Flow Systems, San Diego, CA).

Migration assay

ECs were subjected to electroporation with siRNA and seeded in 6-wells containing EGM2-MV without VEGF. After a regeneration period of 2 h, the cells were washed and the medium was changed to EBM2 basal medium with 0.1% BSA for starvation for 4 h. Cells were harvested and diluted in EBM2 with 0.1% BSA to a final concentration of 2×10^5 per ml. 250 µl were added to the top chamber of a 24-well FluoroBlok™ transwell with 3 µm pore size (Corning Inc., Life Sciences, Tewksbury, MA) and 750 µl of EGM2-MV complete growth medium were added to the bottom well. After an incubation of 22 h the medium was removed and inserts were transferred to a fresh plate containing 500 µl calcein AM solution (4 µg/ml in HBSS) and incubated for 90 min to label migrated cells. The cellular fluorescence was determined in a plate reader with bottom reading capacity (Spectra Max M5, Molecular Devices, Sunnyvale, CA) in scan modus at an excitation/emission wavelength of 485/538 nm. Data were expressed as relative fluorescence units (RFU) or % migration by normalizing to control siRNA treated ECs (value set to 100). Background correction was performed by subtracting the mean fluorescence value of the control well (without ECs) from each sample reading.

Sprouting assay

BECs were transfected with siRNAs and seeded in 6-wells. Six hours after transfection cells were harvested and further diluted to a final concentration of 1.8×10^4 cells per ml M199 medium containing 0.24% ($^w/_v$) methyl cellulose and 10% FBS. Droplets of 25 µl containing approximately 450 cells were applied to a 10x10 cm square plate and incubated upside down over-night. Spheroids were harvested and resuspended in methyl cellulose medium and rat tail collagen (ratio: 1:1) supplied with 10% FBS. The mixture containing approximately 50 spheroids per ml was transferred to a 24-well and left to solidify. After 1 h of incubation at 37°C, 100 µl M199 medium containing the desired stimulus (VEGF at a final gel concentration of 30 ng/ml) was added. The spheroids were incubated for 18 h at 37°C and subsequently fixed with PFA. Bright field images of at least 10 spheroids were acquired for each condition using an Axiovert 40 CFL microscope (Carl Zeiss AG) equipped with a 5×/0.12 A-Plan objective and AxioVision Rel. 4.8 software. Images were processed and cumulative sprout length was calculated using Photoshop CS4 (Adobe Systems Inc.). Live cell imaging of sprouting BECs was carried out with a Cell-IQ® MLF system (CM Technologies, Tampere, Finland) equipped with a Plan Fluor 10×/0.30 DIC objective and with Imagen acquisition software. Z-stacks of bright field images were acquired every 9.5 minutes and rendered to videos with the Cell-IQ® Analyser™ Software. For sprouting assays with cell tracker dyes, the transfected cells were labeled with CellTracker™ Blue CMF_2HC or CellTracker™ Orange CMTMR (Life Technologies, Thermo Fisher Scientific Inc.) by adding the cell dye directly to the cell culture at 5 µM for 30 min prior to spheroid formation. Cells were mixed at equal concentrations. The sprouting assay was carried out as described above. Fixed spheroids were examined with a Zeiss LSM 780 confocal microscope equipped with a 10x objective. Images were obtained and analyzed with Zen2010 software (Carl Zeiss AG) and were further processed with Photoshop CS4 (Adobe Systems Inc.). Only in this instance non-linear adjustment of brightness and contrast were used due to high background fluorescence of the collagen gel.

Mouse retina angiogenesis model

C57Bl/6 wild type littermates were injected at postnatal days P5 and P6 with 50 µg/g 79-6 (Calbiochem – Merck, Darmstadt, Germany) in 100 µl vehicle (peanut oil + 10% ethanol) or with vehicle only. The pups were sacrificed at P7 and the eyes were fixed with 4% PFA for 30 min. Retinas were isolated and stored in methanol at −20°C. For isolectin B4 staining, the retinas were washed with PBS and incubated in 0.5% Triton X-100 in PBS with 1% BSA for 1 h. Retinas were washed twice with PBlec buffer (pH=6.8) containing 1% Triton X-100, 1 mM $CaCl_2$, 1 mM $MgCl_2$ and 0.1 mM $MnCl_2$, and the buffer was replaced by 100 µl PBlec containing 10 µg/ml biotinylated isolectin B4 (Vector Laboratories, Burlingame, CA). Retinas were incubated over-night at 4°C with gentle agitation, then washed with 1% Triton X-100 in PBS and incubated with streptavidin-AlexaFluor555 conjugate (1:500, Invitrogen) over-night at 4°C. Finally, the retinas were washed with PBS, transferred to a microscopic slide and mounted with Fluoromount-G™ (SouthernBiotech). Stained retinas were examined with a Zeiss LSM780 confocal microscope equipped with Plan-Apochromat 5x/0.16 M27 and 10x/0.45 objectives. Tile scans and detailed images of the whole retinas were performed with Zen2010 software (Carl Zeiss AG). Images were further analyzed with either Photoshop CS4 (analysis of branch points and the number of sprouting cells at the angiogenic front) or HistoQuest 3.5 software (TissueGnostics, Vienna, Austria) for the quantification of the isolectin B4 stained vessel area. All experiments were approved by the local ethics committee for animal studies.

Mouse xenograft models of cancer treatment with 79-6

Toledo cells were grown in RPMI 1640 medium with 10% FBS, glutamax and additional 0.25% D-glucose, 1 mM sodium pyruvate and 10 mM HEPES. A total of 1×10^7 cells in 100 µl PBS were injected subcutaneously into the right flank of 8 week old, female CB17/Icr-

Prkdcscid mice (Charles River Laboratories, Wilmington, MA) which were held in a pathogen-free environment. Treatment was initiated 34 days after injection, when tumors had grown to an average size of 57 mm^3. Tumor volume was calculated using the formula (smallest $diameter^2 \times$ largest diameter)/2. Mice were randomized into two groups à 10 animals receiving a daily dose of 50 µg (10 µl) Bcl-6 inhibitor 79-6 or 10 µl of vehicle per gram mouse weight by intraperitoneal injection for 10 consecutive days. The 79-6 compound was dissolved at 50 mg/ml in DMSO, stored at 4^oC for a maximum of 24 h and freshly diluted 1:10 in PBS prior to use. Tumor growth was recorded at 6 time points during treatment.

HT-29 cells were grown in McCoy medium with 10% FBS and 1×10^7 cells in 100 µl PBS were injected subcutaneously into the right flank of 8-10 week old BALB/c/Him nude mice (Division for Laboratory Animal Science and Genetics, Medical University of Vienna, Himberg, Austria). Treatment started on the following day. Animals were randomized into two groups of 8 mice which received a daily dose of 50 µg (10 µl) Bcl-6 inhibitor 79-6 or 10 µl of vehicle per gram mouse weight by intraperitoneal injection for 14 consecutive days. Tumor growth was recorded every second day. One day after the last treatment, animals were sacrificed, tumors were excised, embedded in Tissue-Tek O.C.T. compound and stored at -80^oC.

Analysis of tumor microvessel density

Tumor sections of 10 µm were prepared with a Leica CM3050 S cryostat, generating three sections at 30–50 µm distance for each tumor. Tissues were fixed in ice-cold methanol and treated with 2.4 N HCl for 10 min at room temperature. Incubation with primary antibody #550274 directed against the endothelial marker CD31 (Becton Dickinson, Franklin Lakes, NJ) at 1:50 dilution for 60 min was followed by treatment with secondary reagents (Thermo Fisher): donkey anti-rat AlexaFluor488 at 1:800 and TOTO-3 DNA stain at 1:500 dilution for 30 min. Slides were covered with Fluoromount G (SouthernBiotech) and subsequently analyzed with TissueFAXS, an automated multi-channel immunofluorescence detection system (TissueGnostics, Vienna, Austria). The 20×/0.5 objective was selected for acquisition and the following filter sets were applied: FITC filter for detection of AlexaFluor488-stained CD31 and Cy5 filter for the detection of TOTO-3 stained nuclei. Microvessels in tumor tissue were identified and quantitated using an algorithm developed with StrataQuest 5.0 software (TissueGnostics). Briefly, the signal in the FITC channel was extracted to an 8-bit image, followed by a blurring algorithm (Gauss filter) after which a morphological mask (morphological–close: dilate, erode) was applied and a kernel radius defined. Event detection was based on a minimum of 3 distinct regions within each tumor (in tissue sections at 30–50 µm distance) with an average scanned area of 58 mm^2 per tumor. The kernel radius and background threshold settings were adapted for each batch of scanned images. Figure 7 illustrates the automated steps of vessel recognition and quantitation.

Statistical analysis

Group comparisons were based on Student´s *T*-test and were carried out with SPSS 20 software (IBM Corp., Chicago, IL). Boxplots are generally shown without outliers and extreme values to improve resolution.

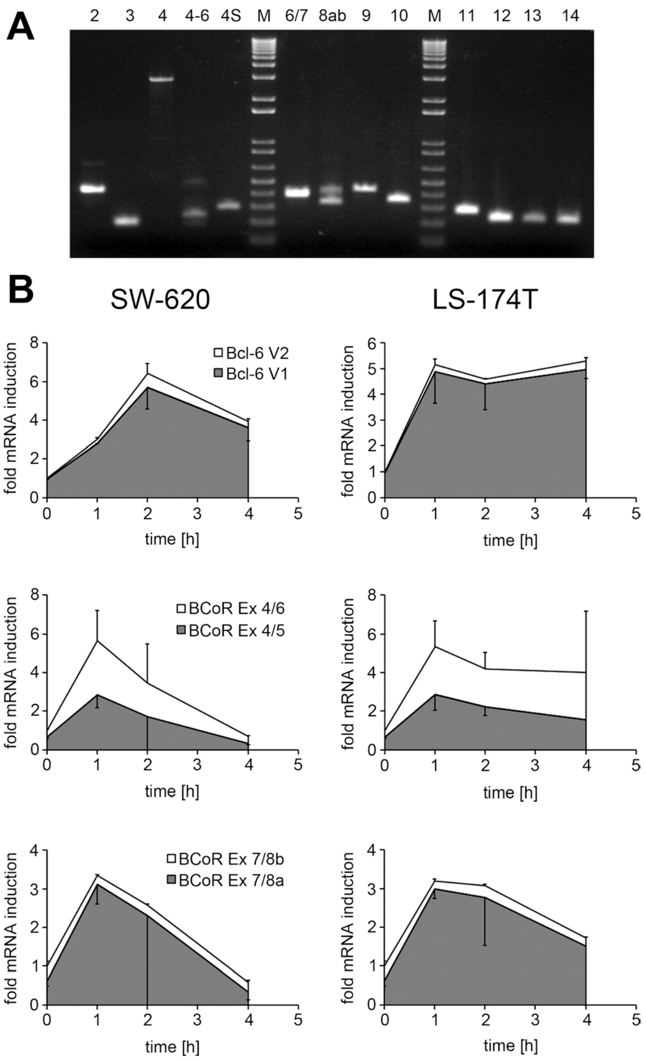

Supplementary Figure S1: Analysis of Bcl-6/BCoR transcript variants. (**A**) The exon structure of BCoR transcripts was evaluated in ECs stimulated with tumor cell supernatant for 2 h. Exon numbers are indicated for each lane. Primer sets were designed to cover entire exons i.e. were placed in adjacent exons to detect alternative splice variants (compare supplementary Table S4). Amplification of exons 4-6 and 8 resulted in 2 bands indicating alternative splicing of exon 5 (4/5 or 4/6) and exon 8a (7/8a or 7/8b). Marker M: 1 kb DNA plus ladder. (**B**) The ratio of Bcl-6 V1/V2 mRNA and of BCoR transcripts with/without exon 5 or exon 8a was assessed by real-time PCR in ECs treated with LS-174T or SW-620 supernatant for 1–4 hours. Specific primer sets were used to distinguish splice variants and the increase in mRNA levels was determined in relation to untreated control. The relative proportion of splice variants within the total amount of transcripts is illustrated by the colors grey and white. The results represent mean values and standard deviations of 2 independent experiments.

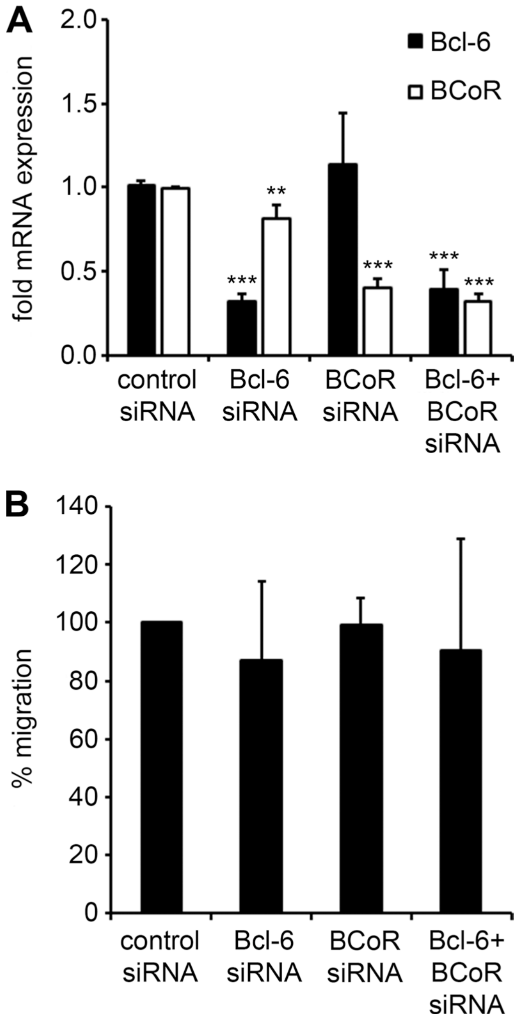

Supplementary Figure S2: Bcl-6/BCoR silencing and its impact on endothelial cell migration. (**A**) Bcl-6/BCoR silencing efficiencies: Cells transfected with Bcl-6 and/or BCoR siRNA were analyzed for transcript levels by real-time PCR at 24 h after transfection. Mean values and standard deviations from 6 independent experiments are shown. (**B**) Migration of endothelial cells with Bcl-6/BCoR silencing was determined using FluoroBlok transwell chambers and is given in relation (%) to ECs transfected with negative control siRNA. The results represent mean values and standard deviations from 3 independent experiments with no significant difference between treatments (*T*-Test; n.s.).

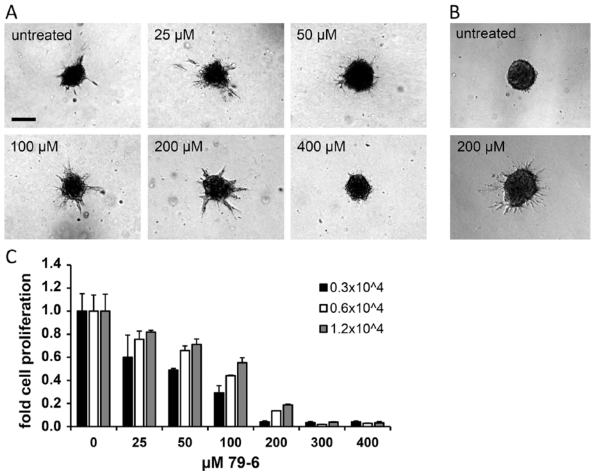

Supplementary Figure S3: The Bcl-6 inhibitor 79-6 induces sprouting and inhibits endothelial proliferation in a dose-dependent manner. (**A**) Human BEC spheroids were embedded in collagen with increasing concentrations of the Bcl-6 inhibitor 79-6. Sprout formation was evaluated after 18 hours. (**B**) Comparably, murine BECs were tested with the optimized 79-6 concentration (200 µM) in a sprouting assay. (**C**) A dose-dependent inhibitory effect of 79-6 on the proliferation of human BECs was confirmed by seeding cells at three different cell densities and measuring DNA synthesis by BrdU incorporation assay.

Supplementary Video S1: Live cell imaging of sprouting BECs. Spheroids of BECs transfected with different siRNAs were embedded in a collagen matrix to monitor sprout formation over 18 h. For control, spheroids were treated with 30 ng/ml VEGF. Live cell imaging was carried out with the Cell-IQ® MLF system, Z-stacks of bright field images were acquired every 9.5 minutes and rendered to videos with the Cell-IQ Analyser Software. See Supplementary_Video S1.

Supplementary Table S1: Microarray analysis of Bcl-6 and co-repressor mRNA expression in endothelial cells exposed to tumor cell derived stimuli

Gene	*GenBank ID*	*Probe Set*	*Exp 1*	*Exp 2*	*Mean*	*SD*
Untreated endothelial cells					***Mean = 1***	
Bcl-6	NM_001706.1	203140_at	351.6	275.3	1.00	0.17
BCoR	NM_017745.1	219433_at	55.7	48.6	1.00	0.10
NCoR	NM_006311.1	200857_s_at	524.9	698.4	1.00	0.20
SMRT	NM_006312.1	207760_s_at	688.7	833.4	1.00	0.13
Stimulated endothelial cells					***mRNA (fold induction)***	
Bcl-6	NM_001706.1	203140_at	360.9	325.6	1.10	0.08
BCoR	NM_017745.1	219433_at	199.7	169.3	3.54	0.41
NCoR	NM_006311.1	200857_s_at	578.4	542.5	0.92	0.04
SMRT	NM_006312.1	207760_s_at	871.4	853.0	1.13	0.02

ECs were seeded in transwells and stimulated with conditioned supernatant from HT-29 (colon carcinoma) cells. Endothelial RNA was harvested after 4 h and analyzed by Affymetrix HU133A microarray for changes in gene expression. Original data (normalized to total gene expression) are given for Bcl-6 and co-repressors BCoR, NCoR, SMRT. Furthermore, mRNA expression of untreated control was set to 1 and changes in transcript levels after EC stimulation were calculated as fold induction (mean and SD of experiment 1 and 2).

Please note that EC stimulations for microarray analysis were conducted in transwells as opposed to regular tissue culture plates in subsequent experiments which showed a faster time course of BCoR mRNA induction.

Microarray data are accessible via the GEO database (accession number GSE67914).

Supplementary Table S2: Sequences of oligonucleotide primers for quantitative real-time PCR with SYBR Green

Gene	*Forward primer*	*Reverse primer*
Bazf	5′-CGGGAAGTGAATTTTTCAGC-3′	5′-TGGTAAGGCTTTTCCCCTGT-3′
Bcl-6	5′-CAAGGCATTGGTGAAGACAA-3′	5′-TGCTATAGAACAGGCCACTGC-3′
BCoR	5′-CCCCCCTTGGCTACGTACT-3′	5′-GCCACTTTCATCATCTGGTTC-3′
BCoR 4/5	5′-GCAAAGAGAATCGCCAACTC-3′	5′-CTCTTGTAATCCTTCCATCTGCAA-3′
BCoR 4/6	5′-GCAAAGAGAATCGCCAACTC-3′	5′-ATTGCACGCTGCAATGCC-3′
BCoR 7/8a	5′-GAGAGGCAGCCTGTGAGC-3′	5′-CTCTCAGGCCAGTCATCTTT-3′
BCoR 7/8b	5′-GAGAGGCAGCCTGTGAGC-3′	5′-ATGGAGCCCTTTAGAGACTCG-3′
Ccna	5′-GATGGTAGTTTTGAGTCACCACA-3′	5′-CACGAGGATAGCTCTCATACTGT-3′
Ccnb	5′-ATAAGGCGAAGATCAACATGGC-3′	5′-TTTGTTACCAATGTCCCCAAGAG-3′
Ccnc	5′-CAGGGCAAGCTGTGTTCCAT-3′	5′-GCAGTGGCAATAACTTGTTGTCT-3′
Ccnd1	5′-GTGCTGCGAAGTGGAAACC-3′	5′-ATCCAGGTGGCGACGATCT-3′
Ccnd2	5′-TGTGCCACCGACTTTAAGTTT-3′	5′-CTTTGAGACAATCCACGTCTGT-3′
Dll4	5′-GCCCTTCAATTTCACCTGGC-3′	5′-CAATAACCAGTTCTGACCCACAG-3′
Hes1	5′-AGCTGGAGAAGGCGGACATT-3′	5′-CGTTCATGCACTCGCTGAAG-3′
Hey1	5′-GAGAACGCCGAAGTGCTGGA-3′	5′-TGGATGTAGCCGGCAGCGAA-3′
Hey2	5′-GAGAAGACTTGTGCCAACTG-3′	5′-TAGGCACTCTCGGAATCCTA-3′
Notch1	5′-GAGGCGTGGCAGACTATGC-3′	5′-CTTGTACTCCGTCAGCGTGA-3′
Notch2	5′-CAACCGCAATGGAGGCTATG-3′	5′-GCGAAGGCACAATCATCAATGTT-3′
Notch3	5′-TGGCGACCTCACTTACGACT-3′	5′-CACTGGCAGTTATAGGTGTTGAC-3′
Notch4	5′-TGTGAACGTGATGTCAACGAG-3′	5′-ACAGTCTGGGCCTATGAAACC-3′

Supplementary Table S3: Sequences of oligonucleotide primers for quantitative real-time PCR with TaqMan probes.
See Supplementary_Table_S3.

Supplementary Table S4: Sequences of oligonucleotide primers for qualitative PCR of BCoR exons

Exon	*Forward primer*	*Reverse primer*	*Amplicon length [bp]*
Exon 2	5′-AACTTTGCTGCTCGCCGCGCTTCTCC-3′	5′-GATCCTATGGGCCGTGCT-3′	430
Exon 3	5′-AGACGACATGCTCTCAGCAA-3′	5′-GATCCTATGGGCCGTGCT3′	199
Exon 4	5′-TGATGGTGACGCTTCAAAAG3′	5′-GAATCGGTCACCCACGTAAC-3′	2830
Exon 4-6	5′-GCAAAGAGAATCGCCAACTC-3′	5′-CAGACACGCCTCAGTGCTAC-3′	193 + 247[b]
Exon 4s[a]	5′-GCAAAGAGAATCGGCCAACTC-3′	5′-CAGACACGCCTCAGTGCTAC-3′	295
Exon 6+7	5′-CCCAGCAACCAAAGACTCC-3′	5′-TAGAGACTCGTCGGCGTTTG-3′	395
Exon 8 a/b	5′-CTCTGCTGAAAGCCAAACG-3′	5′-TGCACTGGTGGATGAAAGAC-3′	243 + 291[c]
Exon 9	5′-CAGAAGCTGGTCGGAAGA-3′	5′-TGCTTGGCTGGTGACAGAT-3′	441
Exon 10	5′-TGCCACTGACAGGGGAATA-3′	5′-GCGTTGTCCCGATGATTTAC-3′	358
Exon 11	5′-CTGGCGAGACCCTTCTGC-3′	5′-GGGGTCAGCACCATAAGAGA-3′	284
Exon 12	5′-CTTGAATATGGCGCTGATGT-3′	5′-CACTGGCGTCATCATCATTG-3′	238
Exon 13	5′-TCAGGTAGAACCATCATGAAAATG-3′	5′-GAAAATTCAAATTCAAACACATCG-3′	242
Exon 14	5′-GGGTCGCAATGATGATGAC-3′	5′-TCCGAAAGCAGTAGCCAGTT-3′	291

[a]4s: exon 4 short; [b]amplicon length without or with exon 5; [c]amplicon length without or with exon 8a.
