# Supplementary material for: Inhibition of the transcriptional repressor complex Bcl-6/BCoR induces endothelial sprouting but does not promote tumor growth
Source: Oncotarget. 2016 Nov 21;8(1):552–64. doi: 10.18632/oncotarget.13477 (PMC5352177; doi:10.18632/oncotarget.13477)
Supplement: Supplementary file 3 [file oncotarget-08-552-s003.doc]

**Table S3.** Sequences of oligonucleotide primers for quantitative real-time PCR with TaqMan probes

| *Gene* | *Forward primer* | *Reverse primer* | *Probe* | *Label* |
| --- | --- | --- | --- | --- |
| *36B4* | 5'-CGCACCGCCGTGATG-3' | 5'-AATAGTTGGATGATCTTAAGGAAGTAGTTG-3' | 5'-AAGACAGGGCGACCTG-3' | Yakima Yellow/BHQ-1 |
| *β-actin* | 5'-CCTGGCACCCAGCACAAT-3' | 5'-GCCGATCCACACGGAGTACT-3' | 5'-ATCAAGATCATTGCTCCTCCTGAGCGC-3' | 6-FAM/BHQ-1 |
| *Bcl-6* | 5'-CAAGGCATTGGTGAAGACAAAAT-3' | 5'-CCGGAGACGATTAAGGTTGAGA-3' | 5'-TCGCCGGCTGACAGCTGTATCCA-3' | Yakima Yellow/BHQ-1 |
| *Bcl-6-V1* | 5'-TGGTGATGCAAGAAGTTTCTAGGAA-3' | 5'-GCCTTGCTTCACAGTCCAAAA-3' | 5'-CACCAGGTTTTGAGCAAAA-3' | Yakima Yellow/BHQ-1 |
| *Bcl-6-V2* | 5'-GTTACAGACTCAAGGAAACCTCTCA-3' | 5'-GCCTTGCTTCACAGTCCAAAA-3' | 5'-TTTGCTCAAAACCAAATGAG-3' | Yakima Yellow/BHQ-1 |
| *BCoR* | 5'-AAGACTCCGAGATGTGCAAATTC-3' | 5'-TACTCGCATCTCTCACTTTCGTTC-3' | 5'-CAGCCGACTGGGAAAGGTTGAAAGG-3' | 6-FAM/BHQ-1 |
